# The Gene Encoding the Antisense Protein ASP of HIV-1: Origin, Distribution and Maintenance

**DOI:** 10.3390/v18030381

**Published:** 2026-03-18

**Authors:** Myriam Abla Houmey, Sara Sadek, Coralie F. Daussy, Nathalie Chazal

**Affiliations:** Institut de Recherche en Infectiologie de Montpellier (IRIM), Université de Montpellier, Centre National de la Recherche Scientifique, 1919 Route de Mende, 34293 Montpellier, Francesara.sadek@irim.cnrs.fr (S.S.); coralie.daussy@irim.cnrs.fr (C.F.D.)

**Keywords:** HIV-1, *asp* gene, overlapping gene, antisense protein, ASP

## Abstract

Human Immunodeficiency Virus Type 1 (HIV-1), the causative agent of the acquired immune deficiency syndrome (AIDS), originated from zoonotic transmissions of simian immunodeficiency viruses (SIVs) infecting African great apes, following complex cross-species transmission events and virus–host co-evolution. These processes were accompanied by multiple viral adaptations, particularly within structural and accessory genes, enabling evasion of host restriction factors and long-term viral persistence. In 1988, an antisense open reading frame (ORF) overlapping the *env* gene was proposed and subsequently confirmed by the identification of antisense transcripts and the antisense protein (ASP). An “intact” ASP ORF (defined as >150 codons) is predominantly conserved in pandemic HIV-1 group M viruses and shows evidence of positive selection, suggesting a selective advantage. Increasing evidence supports the hypothesis that the *asp* gene emerged *de novo* during the evolution of group M and contributed to viral adaptation and global spread in humans. This review combines a narrative review of the literature with original in silico analyses of HIV-1 and SIV sequences retrieved from the Los Alamos National Laboratory database. We systematically reassessed the distribution, length variability and conservation of the ASP ORF across HIV-1 groups (M, N, O, P), subtypes, circulating recombinant forms (CRFs), unique recombinant forms (URFs) and related SIV lineages. Our updated analyses confirmed the strong association between the presence of an “intact” ASP ORF and pandemic HIV-1 group M lineages, while revealing rare but notable antisense ORFs in selected SIVcpz and SIVgor strains. By integrating evolutionary, epidemiological and sequence-based evidence, we aim to clarify the origin and maintenance of the ASP ORF and to contextualize its emergence within the broader framework of overlapping gene evolution, *de novo* gene birth and the selective pressures shaping viral fitness and pandemic potential.

## 1. Introduction

**Figure 1:**
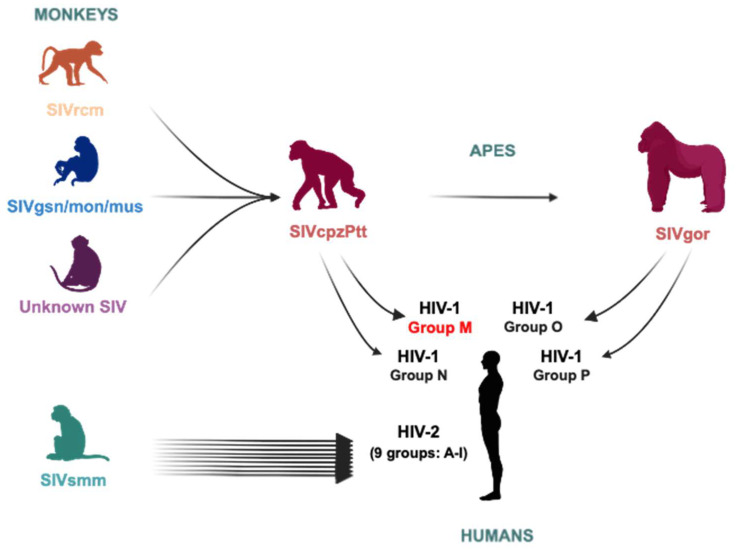
Cross-species transmission events leading to the emergence of HIV-1 and HIV-2 and the determinants governing their zoonotic transfer. The figure was created by NC using BioRender (https://www.biorender.com) 25 July 2025.

Since the discovery of the Human Immunodeficiency Virus type 1 (HIV-1), the causative agent of acquired immune deficiency syndrome (AIDS) [1], its origin has been intensely investigated. At least four independent zoonotic transmission events of simian immunodeficiency viruses from African great apes, specifically chimpanzees (SIVcpz) and gorillas (SIVgor) to humans, gave rise to the four HIV-1 groups: M (“Major”), N (“Non-M Non-O”), O (“Outlier”) and P [2]. These apes themselves acquired SIV infections through earlier cross-species transmission events involving various African monkey species (Figure 1). Among the four HIV-1 groups, group M appears to be the earliest and is responsible for the global pandemic, while groups O and N caused more geographically restricted epidemics. The emergence of group P remains difficult to date due to the limited number of available sequences (only two have been identified so far). Phylogenetic analyses indicate that groups M and N are closely related to SIVcpz strains from southern Cameroon, while groups O and P derive from SIVgor lineages circulating in western and south-central Cameroon [3,4].

The global HIV-1 pandemic, currently affecting more than 40.8 million individuals (UNAIDS, data 2025) is predominantly caused by the pandemic group M (accounting for >98% of all human infections). The epidemiological success of group M has been linked to multiple evolutionary events including repeated cross-species transmissions, recombination events and adaptation to the human host. During this process substantial changes occurred in structural and accessory genes (such as *capsid*, *Vif*, *Vpr*, *Vpx*, *Vpu* and *Nef*) enabling the virus to counteract host restriction factors and optimize replication in human cells [5]. Group M is highly diverse comprising 10 subtypes (A-D, F-H, J, K and L) with multiple sub-subtypes (A1-A8, F1-F2), each exhibiting distinct geographical distributions [6]. In addition, HIV-1 group M includes 167 circulating recombinant forms (CRFs) defined as recombinant HIV-1 genomes identified at least in three epidemiological unrelated individuals (Los Alamos SIV/HIV Sequence Database. http://www.hiv.lanl.gov/content/index accessed 11 July 2025) [7]. (Notably, no sequences are currently available for CRF30_0206; CRF139_02B; CRF142_BC; CRF144_07C; CRF148-cpx; CRF170_0107, CRF172_0755. For CRF168_0107, only one partial sequence has been described, while CRF80_0107 and CRF84-A1D contain non-functional sequences in the *env* gene). CRFs result from recombination events between two or more subtypes or are composed of segments from two or more previously characterized CRFs (so-called second-generation recombinants [7,8]). Unique recombinant forms (URFs) generated by recombination between different subtypes, identified in single individuals or limited cases without evidence of onward transmission, have also been documented.

Between 2016 and 2021, subtype C accounted for 50.4% of global HIV infections, followed by subtype A (12.4%), subtype B (11.3%), subtype G (2.9%), subtype D (2.6%) and subtype F (0.9%) [9]. Subtypes H, J, K and L each contributed 0.1%, while CRFs represented 15.1% of cases, URFs 2.0% and unspecified recombinants 2.2%. Compared to the 2010–2015 period, notable increases were observed in subtype A (+0.9%), subtype C (+3.4%) and unspecified recombinants (+0.5%). In contrast, decreases were recorded for subtype D (−0.5%), subtype G (−0.8%), CRFs (−1.0%) and URFs (−1.8%), while no significant changes were reported for subtypes B and F [9]. The diversity in the global distribution and prevalence of HIV-1 group M subtypes may reflect underlying genomic variation potentially including the emergence and maintenance of additional genetic elements such as the *asp* gene.

**Figure 2:**
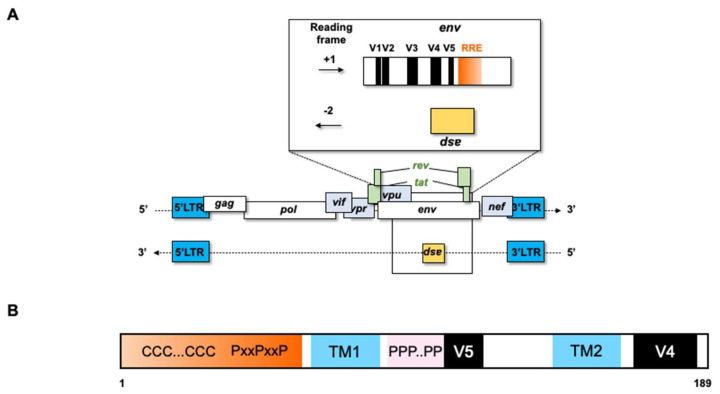
(**A**) Schematic representation of the *asp* gene within the HIV-1 proviral genome. The ASP open reading frame (ORF) overlaps the *env* gene in the -2 reading frame, spanning the hypervariable V4 and V5 regions of *env* and partially overlapping the Rev-Responsive Element (RRE). (**B**) Schematic representation of ASP. The protein contains conserved two cysteine triplets (CCC), one proline-rich SH3-binding motifs (PxxPxxP), one proline rich domain and two predicted transmembrane (TM) domains [12].

An open reading frame (ORF) overlapping the *env* gene in the −2 frame on the antisense strand of the HIV-1 genome, encoding a 189-residue protein, was first proposed in 1988 by R.H. Miller [10] (Figure 2A). Although initially controversial, subsequent studies confirmed the existence of antisense transcripts corresponding to this ORF and demonstrated expression of the antisense protein, ASP [11,12,13,14,15]. Antisense transcripts corresponding to the ASP ORF have been identified in HIV-1-infected cells and the ASP protein has been detected ex vivo, as well as in freshly or chronically HIV-1-infected cells [11,12,13,16,17,18,19,20,21,22,23]. ASP is a small, strongly hydrophobic protein consisting of 189 amino acids in the reference HXB2 sequence (GenBank: K03455). It contains two cysteine triplets (positions 10–12 and 22–24), two proline-rich motifs (positions 47–53 and 101–103) and two strongly hydrophobic putative transmembrane (TM) domains (positions 63–84 and 146–167) (Figure 2B). To date, direct evidence of ASP expression in vivo remains unavailable, primarily due to its inherently low expression levels and biological properties that hinder detection. Furthermore, the functional role of ASP in HIV-1 replication and dissemination remains poorly understood. Nevertheless, indirect evidence supports its expression, as both humoral and cell-mediated immune responses targeting ASP have been observed in HIV-1-infected individuals [24,25,26].

Previous evolutionary analyses have suggested that an “intact” *asp* gene emerged concomitantly with pandemic HIV-1 group M (the term “intact” *asp* gene refers to an Asp coding sequence (ORF) strictly longer than 150 codons) and has been selectively maintained within this lineage [27]. Notably, truncated *asp* variants also occur, and contain one or more premature stop codons. Two recent studies have further supported the hypothesis proposed by Cassan et al. [27]. The first showed that evolutionary constraints act to preserve the ASP amino acid sequence within HIV-1 group M, suggesting functional relevance [28]. The second proposed that the *asp* gene originated *de novo* within HIV-1 group M, rather than being inherited from an ancestral viral genome [29,30]. A subsequent analysis further confirmed that *asp* is a recently created gene within this lineage and likely encodes an accessory protein conferring a selective advantage. More recent large-scale phylogenetic studies have reported correlations between the presence of an “intact” *asp* gene, accelerated disease progression and covariation with gp120 residues implicated in coreceptor usage (CXCR4 or CCR5), suggesting that *asp* may influence viral fitness and tropism [29,30,31]. In addition, incorporation of ASP into virions has been shown to enhance viral entry into CD4^+^ T cells, providing the first direct evidence that ASP contributes to the HIV-1 replication cycle [32].

Despite these advances, important questions remain unresolved. The distribution and integrity of *asp* across the full contemporary diversity of HIV-1 group M, including subtypes, CRFs, and URFs, have not been comprehensively reassessed using updated sequence datasets. Moreover, the evolutionary relationship between *asp* in HIV-1 and potential precursor ORFs in non-pandemic HIV-1 groups (N, O, P) and related simian lineages (SIVcpz and SIVgor) has not been examined within a unified comparative framework. Addressing these issues is essential to determine whether *asp* represents a lineage-specific innovation tightly linked to pandemic emergence or a broader feature of primate lentiviral evolution.

This review therefore analyzes the evolutionary origin of the *asp* gene, its distribution across HIV-1 groups and related primate lentiviruses and the selective forces underlying its maintenance. By integrating comparative genomic and phylogenetic evidence, we aim to clarify how the emergence of this overlapping gene fits within the broader processes of lentiviral genome innovation, host adaptation and pandemic spread.

## 2. Materials and Methods

### 2.1. Sequence Retrieval and Inclusion Criteria

HIV-1 and SIV nucleotide sequences encompassing the *env* gene were retrieved from the Los Alamos National Laboratory (LANL) HIV/SIV Sequence Database (https://www.hiv.lanl.gov/content/index; accessed 11 July 2025). Only sequences covering the full-length *env* region and including the antisense ASP genomic coordinates were included. Partial *env* fragments that did not fully span the ASP antisense region were excluded from prevalence analyses. Sequences annotated as defective or containing large deletions or obvious sequencing artifacts were excluded. When multiple sequences from the same patient were available, only one representative sequence was retained to avoid over-representation.

### 2.2. Identification of Antisense Open Reading Frames

The antisense open reading frame (ORF) was systematically analyzed in the antisense reading frame shifted relative to *env*. ORF detection and sequence analyses were performed using Unipro UGENE [33]. Each sequence was examined individually to identify canonical ATG start codons and in-frame stop codons on the antisense strand. Alternative in-frame start codons located upstream or downstream of the canonical initiation site were also taken into account when defining continuous ORFs.

### 2.3. Operational Definition of Intact and Truncated ASP ORFs

An “intact ASP ORF” was operationally defined as an antisense ORF longer than 150 codons without internal stop codons. ORFs shorter than 150 codons were classified as “truncated ASP ORFs.” This threshold is consistent with previous studies [27].

### 2.4. Sequence Alignment and Motif Verification

Multiple sequence alignments of predicted ASP amino acid sequences were performed within UGENE (version 33) using the ClustalW algorithm with default parameters, followed by manual inspection to confirm motif conservation.

### 2.5. Statistical Considerations

All analyses presented in this study are descriptive in nature. No formal statistical hypothesis testing was performed; therefore, references to “associations” or “correlations” reflect observed distribution patterns rather than statistically inferred relationships.

## 3. Results

### 3.1. Uncovering the ASP ORF in the HIV-1 Groups and Subtypes

**Figure 3:**
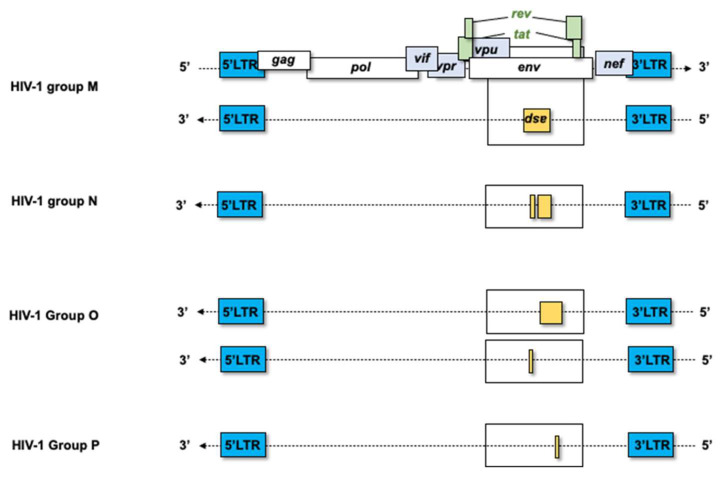
Schematic representation of the *asp* gene within the HIV-1 proviral genome. The ASP open reading frame (ORF) is located on the antisense strand and overlaps the *env* gene in an alternative reading frame. The relative position of some ASP ORFs is shown for HIV-1 groups O, N and P.

As previously discussed, the ASP ORF is thought to have emerged concomitantly with the pandemic HIV-1 group M [27]. To further assess the prevalence and variability of the ASP ORF, we generated new data to complement previously published findings [27]. Following established methodology, we retrieved and analyzed a large number of HIV-1 subtype and sub-subtype sequences encompassing the env gene from the Los Alamos National Laboratory database (https://www.hiv.lanl.gov/content/index accessed 11 July 2025). As in previous studies, our analysis focused on “intact” ASP ORFs longer than 150 codons, while also carefully examining the occurrence of shorter ASP ORFs. Before focusing specifically on HIV-1 group M, we first examined all available sequences from the non-pandemic HIV-1 groups (N, O and P). Although we retrieved a greater number of sequences compared to the original study by Cassan et al. (92 and 12 sequences for groups O and N, respectively, versus 44 and 7), no “intact” ASP ORFs were detected in these groups, consistent with previous reports (Table 1) [27]. Interestingly, truncated ASP ORFs were identified. As illustrated in Figure 3, the asp gene is located on the antisense strand of the HIV-1 proviral genome and overlaps the env gene in an alternative reading frame, and the relative positions of representative ASP ORFs identified in groups O, N and P are indicated. In HIV-1 group N, which is phylogenetically closely related to SIVcpz, 11 sequences exhibited unique shortened ASP ORFs ranging from 130 to 138 codons in length. Additionally, one strain harbored two distinct ORFs (88 and 37 codons, respectively) (Figure 3 and Figure S1A). Alignment of the predicted protein sequences derived from these antisense ORFs with the HIV-1 reference ASP (HXB2, GenBank: K03455) revealed that group N sequences retained key conserved motifs, including cysteine triplets, proline-rich regions, the first transmembrane domain (TM1) and the proline rich motif. Notably, both putative proteins derived from the two ORFs of the strain 02CM-SJGddd (GenBank: GQ324959) displayed a notable degree of conservation (Figure S1A).

Similarly, in HIV-1 groups O and P, which are more closely related to SIVgor, truncated ASP ORFs were identified, ranging from 34 to 134 codons in group O and 76 to 79 codons in group P (Figure 3 and Figure S1B). For group O, three predicted protein sequences were aligned with the HXB2 ASP reference, corresponding to the two longest antisense ORFs (130 and 132 amino acids) and one intermediate-length sequence (72 amino acids) (Figure S1B). For the group P strains, two potential ASP sequences derived from ORFs of respectively 76 and 79 amino acids were similarly analyzed (Figure 3 and Figure S1B). These comparisons revealed also a notable conservation of characteristic motifs: cysteine motif and proline rich motif. Moreover, the two longest sequences from group O and both sequences from group P displayed N-terminal extensions, likely resulting from the use of upstream alternative initiation codon, while the smallest antisense ORF identified in the 99CMA119 strain (GenBank: AF383236) displays a start codon located downstream of the canonical start site.

We then shifted our focus to the pandemic group M and observed considerable variability in the presence of the “intact” ASP ORF across its subtypes (Table 2). Overall, “intact” ASP ORFs were detected in 75.3% of group M sequences (4760/6325). Notably, the most prevalent subtypes C, A, B and G, which together account for 77% of group M infections (with prevalence of 50.4%, 12.4%, 11.3% and 2.9%, respectively; data on CRFs and URFs will be analyzed in a following section) exhibited high frequencies of “intact” ASP ORFs with 77.6% (4576/5898) of their sequences containing this ORF. In contrast, rarer subtypes, such as D, F, J, H, K and L, which collectively account for ∼3.6% of group M infections, showed a significantly lower prevalence of “intact” ASP ORFs, with fewer than 41.5% (174/419) of their sequences harboring the ORF [9]. A more detailed analysis at the sub-subtype level revealed important disparities (Table 3). HIV-1 group M includes eight recognized sub-subtypes (A1, A2, A3, A4, A5, A6, F1, F2) [6,35,36], which are considered distinct lineages, but are not sufficiently divergent to qualify as independent subtypes. Among the globally circulating sub-subtypes of subtype A (A1–A5, A7 and A8), “intact” ASP ORFs were found in 72.7% of sequences (494/679), compared to 66.7% for subtype A overall. Sub-subtypes A1 and A6, both highly prevalent, exhibited the highest frequencies of “intact” ASP ORFs: 64.8% (238/367) and 85.3% (249/292), respectively. Sub-subtype A1 is predominant in East Africa (e.g., Kenya, Uganda, Tanzania), whereas A6 is currently the most rapidly expanding HIV-1 variant in Eastern Europe, particularly in Russia and former Soviet Union [37]. Other sub-subtypes, including A2, A3, A4 and A8, were less prevalent and displayed lower frequencies of “intact” ASP ORF presence: 50%, 25%, 0% and 0%, respectively. A7 was an exception, with 66.7% of its sequences containing the ORF. Interestingly, sequences from sub-subtypes A1, A2, A3, A5, A6 and A7, as well as some A recombinants, harbored an early stop codon shortly after the canonical start site. These sequences featured an alternative in-frame start codon located at amino acid position 26 of the ASP ORF, resulting in truncated ORFs that still exceeded 150 codons in length and likely encoding a shorter form of ASP [27].

Regarding sub-sub-types F1 and F2 (both with low prevalence) (Table 4), the overall frequency of “intact” ASP ORF detection was 40.4% (58/142) (and 100% for the sub-type F: 1/1). Specifically, 37.4% of F1 sequences (46/123) contained the ASP ORF, compared to 61.1% for F2 (11/18). Notably, in addition to the classical “intact” ASP ORF of 150–190 codons and the truncated ASP ORFs found in group M subtypes strains, some HIV-1 strains from group M possess unusually long ORFs exceeding 200 codons: subtype A2 (01CM.1445MV, GenBank: GU201516), subtype D (20941v02_01_7days, GenBank: OM825104; ASP ORF with an alternative in-frame start codon) and subtype G (11439_C5).

GenBank: KR051426; ASP ORF with an alternative in-frame start codon leading to an N-terminal extension in the potential ASP sequence). Moreover, some HIV-1 strains from group M contain not just one, but two truncated ASP ORFs per strain. Examples include subtype D (A08483M1.VRC09A, GenBank: MK501564; ASP ORFs = 109 and 62 codons), subtype F1 (X3016_2s_nfl, GenBank: KJ883143; ASP ORFs = 115 and 49 codons) and subtype F2 (LB002_1, GenBank: MK086130; ASP ORFs = 158 and 47 codons).

### 3.2. Detection of the ASP ORF in HIV-1 CRFs and URFs

The global diversification of HIV-1 has led to the emergence of numerous CRFs and URFs, which together now constitute the second most prevalent group of HIV-1 variants after subtype C. In our analysis of 158 CRFs, the “intact” ASP ORF was detected in 73.4% (2101/2863) of weighted sequences (Table 5 and Table S1), indicating a widespread distribution among recombinant strains. Notably, “intact” ASP ORF was found in 89.2% of CRF01_AE sequences (1308/1466) and 88% of CRF07_cpx sequences (117/133), but in only 5.8% of CRF02_AG sequences (19/328). These three CRFs are among the most epidemiologically significant, with prevalence rates of 5.4%, 1.2% and 5.6% respectively [9]. It is worth highlighting that CRF01_AE, traditionally regarded as a recombinant between subtypes A and E and widely circulating in Asia, has never been linked to a “pure” full-length subtype E genome. Instead, its clusters with unclassified recombinant strains in *env*-based phylogenies [7,39]. CRF02_AG, common in West and Central Africa as well as Taiwan, clusters within subtype A in the *env* region, whereas CRF07_cpx exhibits a subtype C *env*, consistent with its partial subtype C origin [35,36,40]. Further examination of the CRFs landscape revealed substantial heterogeneity in “intact” ASP ORF presence. Specifically, 20 CRFs exhibited low frequencies of ASP ORF detection (≤20% of sequences), including CRF09_cpx, CRF10_CD, CRF12_BF, CRF18_cpx, CRF19_cpx, CRF36_cpx, CRF41_CD, CRF42_BF1, CRF50_A1D, CRF63_02A6, CRF93_cpx, CRF94_cpx, CRF95_02B, CRF105_0108, CRF128_07B, CRF129_56G, CRF130_A1B, CRF131_A1B, CRF132_94B and CRF134_0107. Another 14 CRFs showed moderate prevalence of the ORF (>20–50%), including CRF04_cpx, CRF27_cpx, CRF35_A1D, CRF38_BF1, CRF40_BF1, CRF46_BF1, CRF56_cpx, CRF57_BC, CRF64_BC, CRF72_BF1, CRF96_cpx, CRF99_BF1, CRF124_pcx and CRF158_0107. In contrast, 120 CRFs harbored the ASP ORF in more than half of their sequences, and in 80 of these, an “intact” ASP ORF was identified in 100% of available genomes (Table 5 and Table S1), suggesting a strong and stable integration of “intact” ASP ORF within recombinants forms. Interestingly, some sequences harboring two ASP ORFs are also presented in the CRFs (examples: CFR08_BC: mSSDU91, GenBank: KU820846, ASP ORFs = 11 codons and 52 codons) as well as sequences possessing unusually long ORFs with a canonical start codon exceeding 200 codons (CRF28_BF1: BREPM12313, GenBank: DQ085872; CRF44_BF1: CH12, GenBank: AY536235; CRF88_BC: 05YNRL08sg, GenBank: KC898976; CRF108_BC: Z0230, GenBank: MN172225) or long ORFs with a non-canonical start codon exceeding 200 codons (all the CRF_BG sequences; CRF23_BG: CB118, GenBank: AY900571, AY900572; CFR24_BG: 04BR142, GenBank: AY727527; CRF31_BC: 04BR142, GenBank: AY727527; CRF66_BF1: X4352_2, GenBank: MK298150; GA922739, GenBank:OK011532; CRF71_BF1: 10BR_PE009, GenBank: KJ849760; 10BR_PE088, GenBank: KJ849776; CRF119_0107: nj271, GenBank: MT347592; CRF127_07109: SP233, GenBank: LC735412, CRF152_DG: AF5, GenBank: OR345470; all the available CRF162_cpx sequences). Finally, for URFs, which represent approximately 2.0% of circulating HIV-1 variants [9], the “intact” ASP ORFs were detected in 35.7% of analyzed sequences (10/28). Together, these findings reveal a striking pattern: the “intact” ASP ORF is present in 75.3% of group M sequences (4760/6325). The “intact” ASP ORF is predominantly conserved among subtypes and CRFs with high global prevalence, whereas it is rare or absent in lower-prevalence variants and entirely undetected in the analyzed sequences of non-M groups (N, O and P). This strong association between the presence of the “intact” ASP ORF and pandemic HIV-1 lineages suggests that ASP may contribute to viral fitness, transmissibility, or pathogenicity, thereby supporting its potential role in the global spread and persistence of group M viruses.

### 3.3. Tracking the Emergence of the ASP ORF

The emergence of HIV-1 is rooted in a long evolutionary history of cross-species transmissions of simian immunodeficiency viruses (SIVs) (Figure 1). These events have shaped the genetic landscape of modern primate lentiviruses. SIVs form a highly heterogeneous group infecting over 45 species of African non-human primates, reflecting both their ancient co-divergence with hosts and their remarkable plasticity in adapting to diverse immunological environments [2,41]. Phylogenetic and molecular clock analyses have shown that both pandemic and non-pandemic HIV-1 groups emerged independently from zoonotic transmissions of SIV strains from central chimpanzees (*Pan troglodytes troglodytes*) and western lowland gorillas (*Gorilla gorilla gorilla*) [7,42]. Two distinct SIVcpz lineages have been described, each associated with a different chimpanzee subspecies: SIVcpzPtt in *P. t. troglodytes* and SIVcpzPts in *P. t. schweinfurthii* [43] Notably, only SIVcpzPtt has demonstrated the capacity to cross into humans. Two such transmission events gave rise to HIV-1 group M, the source of the global AIDS pandemic and HIV-1 group N, a rare lineage limited to a few cases in Cameroon [2,44]. The most recent common ancestor (MRCA) of group M is estimated to have emerged around 1908 (range: 1884–1924), while group N likely appeared around 1960 (range: 1940–1980). No zoonotic transmission has been observed for SIVcpzPts to date, possibly due to biological or ecological barriers. Additionally, a transmission of SIVcpzPtt to western gorillas led to the emergence of SIVgor, which later crossed into humans on two separate occasions, giving rise to HIV-1 groups O and P. The MRCA of group O is dated to the 1920s–1930s, and that of group P to the 1940s–1950s [42].

As we did above, we analyzed all available SIVcpz (*n* = 23) and SIVgor (*n* = 6) sequences to assess the presence of antisense ORFs; however, the limited size of this sample necessarily constrains the robustness of the inferences that can be drawn. Our analysis focused specifically on “intact” ASP ORFs strictly longer than 150 codons (Table 6). Among the SIVcpz, comprising 15 SIVcpzPtt and eight SIVcpzPts genomes, we identified antisense ORFs ranging from 41 to 150 codons. Only one SIVcpzPtt strain (MB66, GenBank: DQ373063), isolated in southern Cameroon and closely related to HIV-1 group M contains an “intact” ASP ORF of 181 codons (Table 7; the ASP ORF of MB66, GenBank: DQ373063 is located between reference *env* position 1768–1226) consistent with previous reported by Cassan et al. [27].

In the SIVgor dataset, two of six sequences exhibited truncated antisense ORFs ranging from 42 to 79 codons and three sequences possessed two antisense ORFs within the same sequence (CP2139, GenBank: FJ424864) (Table 7). Surprisingly, one strain (BQID2, GenBank: KP004991) which had not been analyzed in the study by Cassan et al. and closely related to HIV-1 group O was found to harbor an antisense ORF of 175 codons (Table 7; the Antisense ORF of BQID2, GenBank: KP004991 is located between reference *env* position 1771–1247) [45].

Alignment of the predicted protein sequences derived from ORFs from SIVcpzPtt MB66 and SIVgor BQID2 against the HIV-1 reference ASP sequence (HXB2, GenBank: K03455; the ASP ORF is located between reference *env* position 1717–1151) yielded similarity and identity scores of 74.84% and 65.13% for SIVcpzPtt, and 59.30% and 50.75% for SIVgor, respectively. Importantly, conserved motifs of functional relevance, including cysteine triplets, proline-rich domains, and transmembrane helices TM1 and TM2, were substantially retained in these sequences (Figure S2A,B). Moreover, alignment of the predicted protein sequences derived from all ORFs of SIVcpzPtt and SIVgor revealed the conservation of characteristic motifs, and in certain cases, a start codon positioned downstream of the canonical initiation site as well as two antisense ORFs within the same sequences (SIVcpz GenBank: Gab4, GQ217539; LB7, DQ373064; MT145, JN835462 and SIVgor GenBank: CP2139_1con, FJ424864) of which the derived putative protein showed a substantial degree of conservation. Notably, alignment of the predicted protein sequences derived from all ORFs of SIVcpzPts (Figure S2A) revealed antisense ORFs that are generally shorter than those found in SIVcpzPtt sequences, and suggested the possible use of an alternative upstream start codon relative to the canonical one.

### 3.4. Hypotheses on the Origin of the Intact ASP ORF

All these findings, within the context of repeated viral emergence events, raise intriguing evolutionary questions regarding the origin of the “intact” ASP ORF. To date, two mutually exclusive hypotheses have been proposed. One hypothesis suggests that ASP represents an ancestral ORF present in a common primate lentiviral ancestor and subsequently lost in most lineages except the HIV-1 group M [30]. The second hypothesis, more widely supported, proposes that *asp* is a recent genetic innovation, a *de novo* gene that specifically emerged during the evolution of HIV-1 group M following its divergence from SIVcpzPtt [27,46]. Several lines of evidence support the *de novo* emergence hypothesis. First, exhaustive genomic analyses of SIVcpz and non-pandemic strains of HIV-1 (groups N, O and P) reveal no “intact” ASP ORF, suggesting that the *asp* gene emerged after the divergence of group M. Second, bioinformatic and evolutionary analysis of the nucleotide and codon sequence of the antisense ORF of HIV-1 strains belonging to group M show the creation of a start codon, a progressive elimination of internal stop codons and the evolution of a nucleotide sequence that reduces the likelihood of new stop codons emerging from synonymous mutations in *env* [28,46]. Third, the high frequency of “intact” ASP ORFs in various pandemic strains of group M suggests a selective advantage [11,27]. However, the presence of an “intact” ASP ORF in the MB66 strain of SIVcpz, as well as in the BQID2 strain of SIVgor, both potentially encoding antisense proteins that retain conserved functional motifs, raises additional questions (Table 6 and Figure S2). Currently, several hypotheses remain open: did the “intact” ASP ORF emerge broadly among ancestral SIVcpzPtt strains and subsequently persist during the zoonotic emergence and diversification of HIV-1 group M? Since only a limited number of SIVcpzPtt sequences are available, we cannot draw a definitive conclusion. Alternatively, could the “intact” ASP ORF have arisen sporadically within specific SIVcpzPtt strains, conferring a selective advantage facilitating specific events of transmission between chimpanzees and/or cross-species transmission from chimpanzees to humans? Could the ASP ORF have evolved independently in both SIVcpzPtt and HIV-1 lineages? Furthermore, the presence of a potentially ancestral “intact” ASP ORF in one SIVgor strain raises intriguing and questions and possibilities: as SIVgor have acquired SIV from chimpanzees, did the SIVcpzPtt strain that gave rise to SIVgor already possess an antisense ORF? Might this ancestral antisense ORF have facilitated cross-species transmission events from gorillas to humans, akin to those hypothesized for SIVcpzPtt? SIVcpzPtt transmission to western gorillas led to the emergence of SIVgor. If so, did this ORF undergo subsequent evolutionary loss in HIV-1 group O strains, or is its apparent absence merely reflective of incomplete representation in current sequence databases? Looking further back, SIVcpz, transmitted directly from chimpanzees to humans twice and also transmitted once to gorillas [47], has been shown to originate from multiple cross-species transmission events and recombination between different simian lentiviruses. Specifically, it is now established that SIVcpz arose from recombination between viruses infecting greater spot-nosed monkeys (*Cercopithecus nictitans*), mustached monkeys (*Cercopithecus cephus*) and mona monkeys (*Cercopithecus mona*), collectively referred to as SIVgsn/SIVmus/SIVmon, and SIVrcm, which infects red-capped mangabeys (*Cercocebus torquatus*) [48]. Bailes et al. proposed that SIVrcm contributed the 5′ portion of the SIVcpz genome, while the SIVgsn/-mus/-mon lineage provided the 3′ portion [48]. More recently, it has been suggested that the SIVcpz genome may be tripartite in origin. According to this model, the 5′ region could derive from an as-yet unidentified virus, the *pol* and *vif* genes may originate from either SIVrcm or SIVmnd-2, and the 3′ region, encompassing *rev*, *vpu*, *tat*, *env* and *nef*, would stem from the SIVgsn/mon/mus clade [49]. Since “intact” or truncated ASP open reading frame (ORF) are present in SIVcpzPtt sequences and SIVgor, both HIV-1 precursors, we investigate the presence of ancestral antisense ORFs notably in the 3′ portion of the precursor lineages of SIVcpzPtt: SIVgsn/mon/mus. This analysis was not possible in the SIVgor sequences, since most of the SIVgor genome represents a complex recombinant of multiple diverse SIVgor lineages still not identified. Among the 10 available sequences of SIVgsn/mon/mus analyzed, antisense ORFs were identified in all lineages within the *env* region. In SIVgsn short antisense ORFs of 74 and 93 codons were detected in strains 99C166 (GenBank: AF468659; *env* positions 1753–1475) and 99CM71 (GenBank: AF468658; *env* positions 1780–1559), respectively. In contrast, SIVmon sequences (NG1, AJ549283; 99CMCML1, AY340701) harbored longer antisense ORFs (>124 codons) located between *env* positions 1936–1376 and 1822–1451, respectively. Similarly, SIVmus1, 2 and 3 strains contained antisense ORFs ranging from 102 to 192 codons located between the *env* positions 1831–1382 for SIVmus1 (01CM1085, GenBank: AY340700) and 1813–1508-for SIVmus1 (01CM1239, GenBank: EF070330), 1981–1406 for SIVmus2 (01CM1246, GenBank: EF070329) and 1777–1328 for SIVmus2 (01CM2500, GenBank: EF070331) and 1909–1511-for SIVmus3 (09GabOI81, GenBank: KF304707) and 1888–1436 for SIVmus3 (11GabPts02, GenBank: KF304708). Then, we performed alignment of the predicted antisense proteins with HXB2 ASP (GenBank: K03455) together SIVcpz*Ptt* MB66 ASP (GenBank: DQ373063) and SIVgor BQID2 ASP (GenBank: KP004991) (Figure S3). We observed that the two SIV*gsn* sequences (99CM71, GenBank: AF468658 and 99CM166, AF468659) contain truncated ASP-like ORFs (74 and 93 codons, respectively). In contrast, the two SIV*mon* sequences (NG1, GenBank: AJ549283 and 99CMCML1, AY340701) and six SIV*mus* sequences (01CM1085, GenBank: AY340700; 01CM1239, EF070330; 01CM1246, EF070329; 01CM2500, EF070331; 09GabOI81, KF304707 and 11GabPts02, KF304708) harbor antisense ORFs of 187, 124, 150, 102, 192, 150, 133 and 151 codons, respectively. Overall, while SIVgsn appears to possess only short truncated antisense ORFs, several SIVmon and SIVmus strains harbor antisense ORFs ranging from approximately 124 to 192 codons, frequently initiated from upstream alternative start codons. This pattern suggests the presence of a rudimentary ASP-like ORFs in the 3′ parental lineage that contributed to the emergence of SIVcpzPtt [29].

## 4. Discussion

In this study, we performed a comprehensive and updated reassessment of the distribution and evolutionary context of the ASP ORF across the full spectrum of HIV-1 diversity and related simian lentiviruses. Our analyses confirm and extend previous reports indicating the presence of an “intact” ASP ORF (>150 codons) is strongly associated with pandemic HIV-1 group M. 75.3% of group M sequences harbor an “intact” ASP ORF with even higher frequencies among the most epidemiologically successful subtypes and CRFs [27]. When considering the dominant subtypes A, B, C and G together with major CRFs, the prevalence of “intact” ASP ORFs reaches more than 78% representing the vast majority of globally circulating viruses. In contrast, no “intact” ASP ORF was detected in the currently available sequences from non-pandemic HIV-1 groups N, O and P despite the recurrent presence of truncated antisense ORFs. This striking distribution pattern reinforces the concept that stabilization of a full-length ASP ORF is a lineage-specific feature tightly linked to the emergence and global dissemination of group M viruses.

Within group M, however, substantial heterogeneity exists. Nearly one quarter of sequences lack an “intact” ASP ORF, according to the operational threshold > 150 codons. Marked differences are observed between high and low prevalence subtypes. Notably, approximately 24–25% of group M strains and more than 20% of high prevalence subtypes/CRFs harbor truncated ASP ORFs whereas nearly half of low prevalence subtypes retain an “intact” ORF. This variability indicates that ASP seems not strictly essential for virus replication *per se*, but rather behaves as a modulatory accessory factor whose contribution to viral fitness may be context dependent [46,50]. Importantly, this pattern is not unique within HIV-1 genome. The frequency of viruses lacking an “intact” *asp* gene is comparable to that observed in subtype B viruses lacking an “intact” *nef* gene (15% versus 13.5%) [51]. Thus, disruption of accessory genes does not preclude transmission but may modulate pathogenicity, replication efficiency or transmissibility. Increasing evidence from studies of overlapping and *de novo* viral genes indicates the “accessory” does not equate to “dispensable” [52]. Rather, such genes often confer measurable fitness advantages at specific stages of the viral life cycle, including enhanced replication efficiency, modulation of cell tropism, immune evasion and host-to-host transmission [50]. ASP likely belongs to this functional category. Recent functional studies provide biological plausibility for the selective maintenance of ASP in group M. In silico analyses indicate that the presence of an “intact” ASP ORF correlates with faster disease progression and covaries with gp120 residues within the V3 loop associated with coreceptor usage [29,31]. Moreover, experimental evidence demonstrates that virion-associated ASP enhances entry into CD4^+^ T cells, directly implicating ASP in the viral replication cycle and thereby promote transmission [32]. These convergent data suggest that ASP may enhance viral fitness by modulating entry efficiency, tropism or transmission dynamics.

The detection of “intact” antisense ORFs in one SIVcpzPtt and one SIVgor strain introduces important evolutionary considerations. Although rare in our dataset, these ORFs retain conserved ASP features, including cysteine triplets, proline-rich motifs and predicted transmembrane domains. These findings suggest that antisense coding potential may predate HIV-1 group M existing as rudimentary ORFs in ancestral lineages. The identification on antisense ORFs of variable length in SIVgsn, SIVmon and SIVmus lineages further supports the notion that this genomic region represents an evolvable antisense coding reservoir. The hypothesis of *de novo* gene birth in group M remains strongly supported by multiple lines of evidence: (i) the absence of conserved “intact” ASP ORFs in non-M HIV-1 groups; (ii) molecular signatures consistent with progressive removal of stop codons and establishment of functional start codon; and (iii) selective constraints acting on the antisense frame despite its overlap with env gene. These scenarios are not mutually exclusive. Ancestral truncated ORFs may have provided a substrate upon which progressive refinement and selective stabilization occurred during the emergence of group M.

A methodological point deserves clarification. Throughout the original and subsequent evolutionary studies, an “intact” ASP ORF has been operationally defined as an antisense ORF strictly longer than 150 codons. This threshold was initially chosen because ORFs of this size are more likely to encode proteins containing recognizable structural domains, conserved motifs (e.g., cysteine triplets, proline-rich regions), and predicted transmembrane segments, thereby strengthening the argument for functional relevance. However, this cutoff is intentionally conservative and inevitably excludes shorter antisense ORFs that may retain biological activity [53,54,55]. Importantly, shorter ORFs are not rare outside pandemic group M. In non-pandemic HIV-1 groups N, O and P, we did not detect any ORF exceeding 150 codons, yet multiple truncated antisense ORFs were observed, typically ranging from ~34 to 138 codons (groups O and P: 34–134 and 76–79 codons; group N: 130–138 codons). In SIVcpz and SIVgor lineages, antisense ORFs were also frequently present but generally shorter than 150 codons (41–150 codons in SIVcpz; 42–79 codons in most SIVgor strains), with only one SIVcpzPtt strain and one SIVgor strain harboring an ORF exceeding the 150-codon threshold. Similarly, in SIVgsn/mon/mus precursor lineages, antisense ORFs of variable length (approximately 74–192 codons, often initiated from alternative upstream start codons) were identified. Thus, shorter antisense ORFs appear recurrently across primate lentiviral lineages, even when a canonical “intact” ASP ORF is absent. These observations reinforce that the >150-codon definition enriches for long, motif-complete ASP variants characteristic of pandemic group M, but does not imply that shorter forms are biologically irrelevant. Increasing evidence from viral and cellular systems indicates that small proteins can exert regulatory or modulatory functions. Future work should therefore explicitly evaluate the expression and functional relevance of shorter ASP variants, particularly in non-M HIV-1 groups and SIV lineages, using transcript detection, ribosome profiling, proteomics and targeted antibody approaches. A comprehensive assessment of these shorter antisense products will be necessary to determine whether ASP evolution involved gradual extension and stabilization of a pre-existing rudimentary ORF or the selective refinement of multiple size variants.

Taken together, our findings support a model in which antisense coding potential in the *env*-overlapping frame existed in ancestral primate lentiviruses as truncated or unstable ORFs and was subsequently stabilized, extended and selectively maintained during the emergence of pandemic HIV-1 group M. The preferential conservation of a full-length ASP ORF within the pandemic lineage, combined with evidence of selective pressure in the overlapping *env* region is consistent with the hypothesis that ASP may have contributed to the evolutionary success, adaptation and global spread of group M viruses.

Several non–mutually exclusive evolutionary scenarios may explain the current distribution of “intact” ASP ORFs across HIV-1 and SIV lineages. First, an “intact” ASP ORF may have been present in ancestral SIVcpzPtt and subsequently lost in certain descendant lineages. Second, an “intact” ASP ORF may have arisen independently in specific HIV-1 lineages after cross-species transmission, through lineage-specific sequence changes restoring or extending the antisense reading frame. Third, the ASP ORF may have undergone dynamic fluctuations in length and integrity over time, with stabilization occurring only in particular genetic backgrounds under distinct selective pressures. At present, however, the limited number and geographic scope of available SIV genomes do not allow discrimination among these scenarios. Therefore, any inference regarding the ancestral state and evolutionary trajectory of ASP must be considered provisional.

## 5. Conclusions

This study provides an updated evolutionary framework for understanding the origin and maintenance of the *asp* gene. An “intact” ASP ORF is a defining feature of pandemic HIV-1 group M, present in 75.3% of sequences and highly enriched among dominant subtypes and CRFs, yet absent from currently characterized non-pandemic HIV-1 groups. This distribution reveals a strong association between the presence of an “intact” ASP ORF and pandemic lineages.

Although ASP is not strictly required for viral replication, convergent evolutionary epidemiological and experimental evidence supports the hypothesis that it may enhance viral fitness. These findings are consistent with a potential role for ASP in modulating entry efficiency, cell tropism, disease progression or transmission dynamics. Nevertheless, the available data do not demonstrate that ASP emergence *per se* causes the global expansion of group M. The preferential conservation of an “intact” ASP ORF in pandemic lineages may alternatively reflect linkage with other adaptative changes in the overlapping *env* gen or broader genomic contexts that favored transmission and spread.

Beyond HIV-1 biology, the *asp* gene exemplifies overlapping gene evolution and potential *de novo* gene emergence within a highly constrained retroviral genome. Its evolutionary trajectory illustrates how new coding capacities can arise, become refined under selective pressure and contribute to pandemic potential. Future investigations should determine whether truncated ASP variants are expressed in vivo, clarify the structural organization and molecular patterns of ASP and define mechanistic contribution to viral replication and transmission. Elucidating theses aspects will be essential to fully understand the role of ASP in HIV-1 pathogenesis and the broader principles governing viral genome innovation.

## Figures and Tables

**Table 1:** Prevalence in the human population of HIV-1 groups N, O and P [34] and proportion of sequences containing an ASP ORF longer than 150 codons.

HIV-1 GROUPS	SEQUENCES WITH ASP ORF (Length > 150 Codons)	PREVALENCE (2004–2007) [34]
HIV-1 GROUP N	0/12 (0.0%)	~0.0%
HIV-1 GROUP O	0/92 (0.0%)	<0.1%
HIV-1 GROUP P	0/2 (0.0%)	~0.0%
**Total HIV-1 GROUPS N, O, P**	**0/106 (0.0%)**	**~0.0%–<0.1%**

**Table 2:** The table presents the proportion of sequences containing an “intact” ASP ORF (length > 150 codons) corresponding to the number of analyzed *env* sequences with an ASP ORF > 150 codons relative to the total number of sequences analyzed for each subtype. It also includes the prevalence of each subtype, CRFs and URFs in the human population for the HIV-1 group M, subtypes and CRF [9,38]. * The data corresponding to subtypes B and C were reported previously in Cassan et al. [27].

HIV-1 GROUP M	SEQUENCE WITH ASP ORF (Length > 150 Codons)	PREVALENCE (2010–2015) [9,38]	PREVALENCE (2016–2021) [9,38]
A (A,A1,A2,A3,A4,A6)	498/685 (72.7%)	11.5%	12.4%
B	1111/1307 * (85.0%)	11.4%	11.3%
C	728/864 * (84.0%)	47.1%	50.4%
D	106/254 (41.7%)	3.1%	2.6%
F,F1,F2	58/142 (40.8%)	0.8%	0.9%
G	138/159 (86.8%)	3.8%	2.9%
H	1/10 (10.0%)	0.2%	≤0.1%
J	5/7 (71.4%)	0.3%	~0.0%
K	1/2 (50.0%)	0.2%	~0.0%
L	3/4 (75.0%)	ND	ND
Total CRFs	2101/2863 (73.4%)	16.2%	15.1%
UFRs	10/28 (35.7%)	3.8%	2.0%
**Total GROUP M**	**4760/6325 (75.3%)**		

**Table 3:** The table provides the proportion of sequences with the ASP ORF (length > 150 codons) corresponding to the number of analyzed *env* sequences containing an ASP ORF > 150 codons relative to the total number of sequences analyzed for each subtype, as well as the prevalence in the human population of HIV-1 group M subtypes A [9,38].

HIV-1 GROUP M SUBTYPES A	SEQUENCES WITH ASP ORF (Length > 150 Codons)	COUNTRIES
A	4/6 (66.7%)	Democratic Republic of the Congo (DRC), Kenya, Uganda, South Africa, Switzerland.
A1	238/367 (64.8%)	Uganda, Rwanda, Kenya, Tanzania, Sweden, Australia, Cyprus, Spain, Bulgaria, Belgium, Cameroon, DRC, Finland, Gambia, India, Nepal, Pakistan, South Africa, South Korea, United Kingdom, USA.
A2	4/8 (50.0%)	DRC, Cameroon, Cyprus, South-Korea.
A3	1/4 (25.0%)	Senegal, Gambia, USA, France
A4	0/3 (0.0%)	DRC
A6	249/292 (85.3%)	Russian Federation, Ukraine, Belarus, Georgia, Ubezkistan, Kazakhstan, Italy, Cyprus, Slovenia, Belgium, United Kingdom, China, Thaïland
A7	2/3 (66.7%)	Nigeria, Cyprus
A8	0/2 (0.0%)	Cape Verde
**Total A1 to A8**	**494/679 (72.7%)**	
**Total**	**498/685 (72.7%)**	

**Table 4:** The table provides the proportion of sequences with the ASP ORF (length > 150 codons) corresponding to the number of analyzed *env* sequences containing an ASP ORF > 150 codons relative to the total number of sequences analyzed for each subtype, as well as the prevalence in the human population of HIV-1 group M subtypes F [9,38].

HIV-1 GROUP M SUBTYPES F	SEQUENCES WITH ASP ORF (Length > 150 Codons)	COUNTRIES
F	1/1 (100.0%)	Brazil
F1	46/123 (37.4%)	Brazil, Spain, France, United Kingdom, Finland, Russian Federation, Belgium, Roumania, Argentina, Peru, Germany, Angola, Bulgaria
F2	11/18 (61.1%)	Cameroon, Spain, South Africa
Total F1 to F2	57/141 (40.4%)	
**Total**	**58/142 (40.8%)**	

**Table 5:** Proportion of sequences with the ASP ORF (length > 150 codons) corresponding to the number of analyzed *env* sequences containing an ASP ORF > 150 codons relative to the total number of sequences analyzed for each CRF/URF, as well as the prevalence in the human population of HIV-1 group M CRFs and URFs [9,38]. Notably, no sequences are currently available for CRF30_0206; CRF139_02B; CRF142_BC; CRF144_07C; CRF148-cpx; CRF170_0107; CRF172_0755. For CRF168_0107, only one partial sequence has been described, while CRF80_0107 and CRF84-A1D contain non-functional sequences in the *env* gene.

HIV-1 GROUP M CRFs and URFs	SEQUENCES WITH ASP ORF (Length > 150 Codons)	PREVALENCE (2010–2015) [9,38]	PREVALENCE (2016–2021) [9,38]
**TOTAL CRFs**	**2101/2863 (73.4%)**	16.20%	15.10%
**TOTAL UFRs**	**10/28 (35.7%)**	3.80%	2.00%
**Total HIV-1 CRFs+ URFs**	**2111/2891 (73.0%)**		

**Table 6:** Proportion of sequences with the ASP ORF (Length > 150 codons).

SIV	SEQUENCES WITH ASP ORF (Length > 150 Codons)
SIVcpz Ptt	1/15 (6.7%)
SIVcpz Pts	0/8 (0.0%)
**SIVcpz total**	**1/23 (4.3%)**
SIVgor	1/6 (16.6%)
**SIVgor total**	**1/6 (16.6%)**

**Table 7:** Lengths of the ASP ORFs detected in SIVcpz and SIVgor sequences.

SIVcpz GenBank NUMBER	SIVcpz NAME	LENGHT OF THE ASP ORF	CHIMPANZEE SUBSPECIES
AY169968	SIVcpzCAM13	41	Ptt
GQ217539	SIVcpzGab4	69 + 44	Ptt
X52154	GAB1	78	Ptt
DQ373064	SIVcpzLB7	87 + 87	Ptt
JN835462	SIVcpzMT145	89 + 48	Ptt
FR686511	Ptt_09Cam155	90	Ptt
AF382828	SIVcpzGAB2	95 + 52	Ptt
JN835460	SIVcpzEK505	116	Ptt
JN835461	SIVcpzMB897	126	Ptt
AJ271369	CAM5	138	Ptt
AF115393	CAM3	139	Ptt
EF535993	SIVcpzDP943	144	Ptt
JX178450	LB715	147	Ptt
AF103818	US_Marilyn	150	Ptt
***DQ373063***	***SIVcpzMB66***	***181***	***Ptt***
JQ866001	BF1167	48	Pts
JN091690	UG38	53	Pts
EF394356	TAN1	79	Pts
JN091691	TAN5	81	Pts
JQ768416	SIVcpzTAN13	81	Pts
EF394358	TAN3	81	Pts
EF394357	TAN2	99	Pts
U42720	ANT	101	Pts
**SIVgor** **GenBank NUMBER**	**SIVgor NAME**	**LENGHT OF THE** **ASP ORF**	**GORILLA** **SUBSPECIES**
FJ424863	SIVgorCP2135con	42	Gor
FJ424864	SIVgorCP2139_1con	69 + 45	Gor
FJ424871	SIVgorCP684con	79	Gor
KP004989	SIVgor-BPID1	88 + 87	Gor
KP004990	SIVgor-BPID15	89 + 49	Gor
***KP004991***	***SIVgor-BQID2***	***175***	***Gor***

## Data Availability

Data availability requests can be fulfilled by contacting the corresponding author.

## Supplementary Materials

The following supporting information can be downloaded at: https://www.mdpi.com/article/10.3390/v18030381/s1, Figure S1: (A) Amino acid sequence alignment of ASP from the HIV-1 group N isolates (GenBank: GQ324962, JN572926, DQ017382, AY532635, KY953204, AJ271370, DQ017383, AJ006022, KY498771, MF767262, GQ324958, GQ324959) and SIVcpz (GenBank: DQ373063), using HXB2 (GenBank: K03455) as the reference sequence. The HIV-1 sequences were retrieved from HIV-1 data bases, Los Alamos National Laboratory and analyzed using Unipro UGENE: an unified bioinformatics toolkit [33]. (B) Amino acid sequence alignment of the antisense protein (ASP) from HIV-1 group O (GenBank: AF383253, AF383245, AF383236) and P isolates (GenBank: HQ179987, GQ328744) and SIVcpz (GenBank: DQ373063), using HXB2 (GenBank: K03455) as the reference sequence. The main motifs of ASP are indicated; Figure S2: (A) Alignment of the ASP from multiple SIVcpzPtt (GenBank: AF103818, JX178450, EF535993, AF103818, AF115393, AJ271369, JN835461, JN835460, GQ217539, DQ373064, JN835462, AF382828, FR686511, X52154) and SIVcpzPts isolates (GenBank: U42720, EF394357, EF394358, JQ768416, EF394356, JN091690, JQ866001, JN091691) with HXB2 (GenBank: K03455) and SIVcpz (GenBank: DQ373063). The first line represents the reference HXB2 sequence of ASP. The main motifs of ASP are indicated. (B) Sequence alignment of the ASP from SIVgor isolates (GenBank: KP04990, KP04989, FJ424871, FJ424863, FJ424864) compared with HXB2 (GenBank: K03455) and SIVgor (GenBank: KP004991). The first line represents the reference HXB2 sequence of ASP. The main motifs of ASP are indicated; Figure S3: Sequence alignment of the ASP from SIVgsn, SIVmon, SIVmus1, mus2 and mus3 compared with HXB2 (GenBank: K03455), SIVcpz (GenBank: DQ373063) and SIVgor (GenBank: KP004991). The SIVgsn (GenBank: AF468659, AF468658), SIVmon (GenBank: AJ549283, AY340701), SIVmus1 (GenBank: AY340700, EF070330), mus2 (GenBank: EF070329, EF070331) and mus3 (GenBank: KF304707, KF304708) sequences were retrieved from HIV-1 data bases, Los Alamos National Laboratory. The first line represents the reference HXB2 sequence of ASP. The main motifs of ASP are indicated; Table S1: The table provides the proportion of sequences with the ASP ORF (length > 150 codons) corresponding to the number of analyzed *env* sequences containing an ASP ORF > 150 codons relative to the total number of sequences analyzed for each CRF/URF, as well as and the prevalence in the human population of HIV-1 group M CRFs and URFs [9,38]. Notably, no sequences are currently available for CRF30_0206; CRF139_02B; CRF142_BC; CRF144_07C; CRF148-cpx; CRF170_0107; or CRF172_0755. For CRF168_0107, only one partial sequence has been described, while CRF80_0107 and CRF84-A1D contain non-functional sequences in the *env* gene.
