# Supplementary material for: The Gene Encoding the Antisense Protein ASP of HIV-1: Origin, Distribution and Maintenance
Source: Viruses. 2026 Mar 18;18(3):381. doi: 10.3390/v18030381 (PMC13030601; doi:10.3390/v18030381)
Supplement: Supplementary file 1 [file viruses-18-00381-s001.zip › Houmey et al Supplementary Figures and Tables Revised Version February 2026 MARS FINAL PROOF.pptx]

## Slide 1
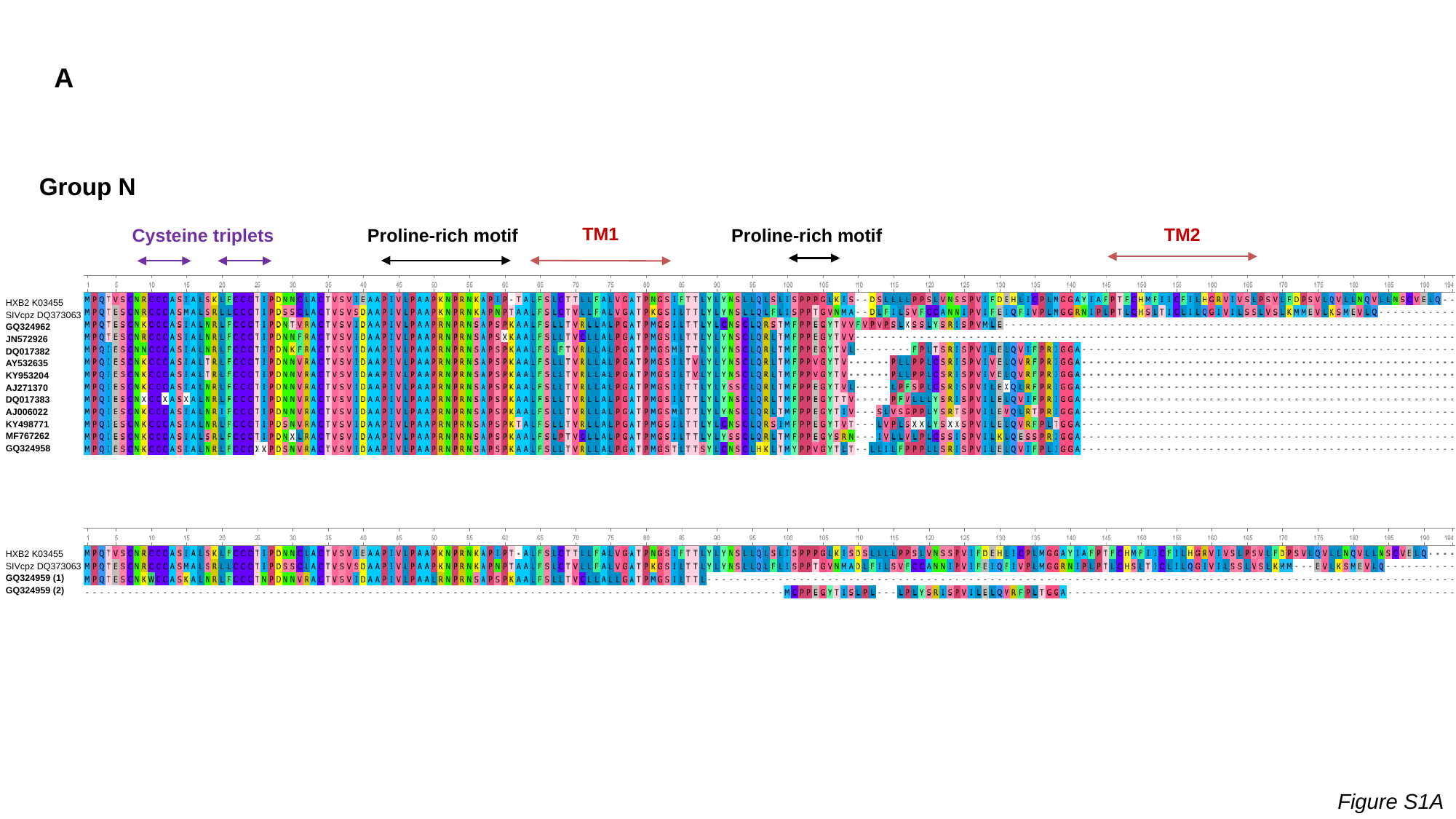

A
Group N
TM1
TM2
Cysteine triplets
Proline-rich motif
Proline-rich motif
HXB2 K03455
SIVcpz DQ373063
GQ324962
JN572926
DQ017382
AY532635
KY953204
AJ271370
DQ017383
AJ006022
KY498771
MF767262
GQ324958
HXB2 K03455
SIVcpz DQ373063
GQ324959 (1)
GQ324959 (2)
Figure S1A

## Slide 2
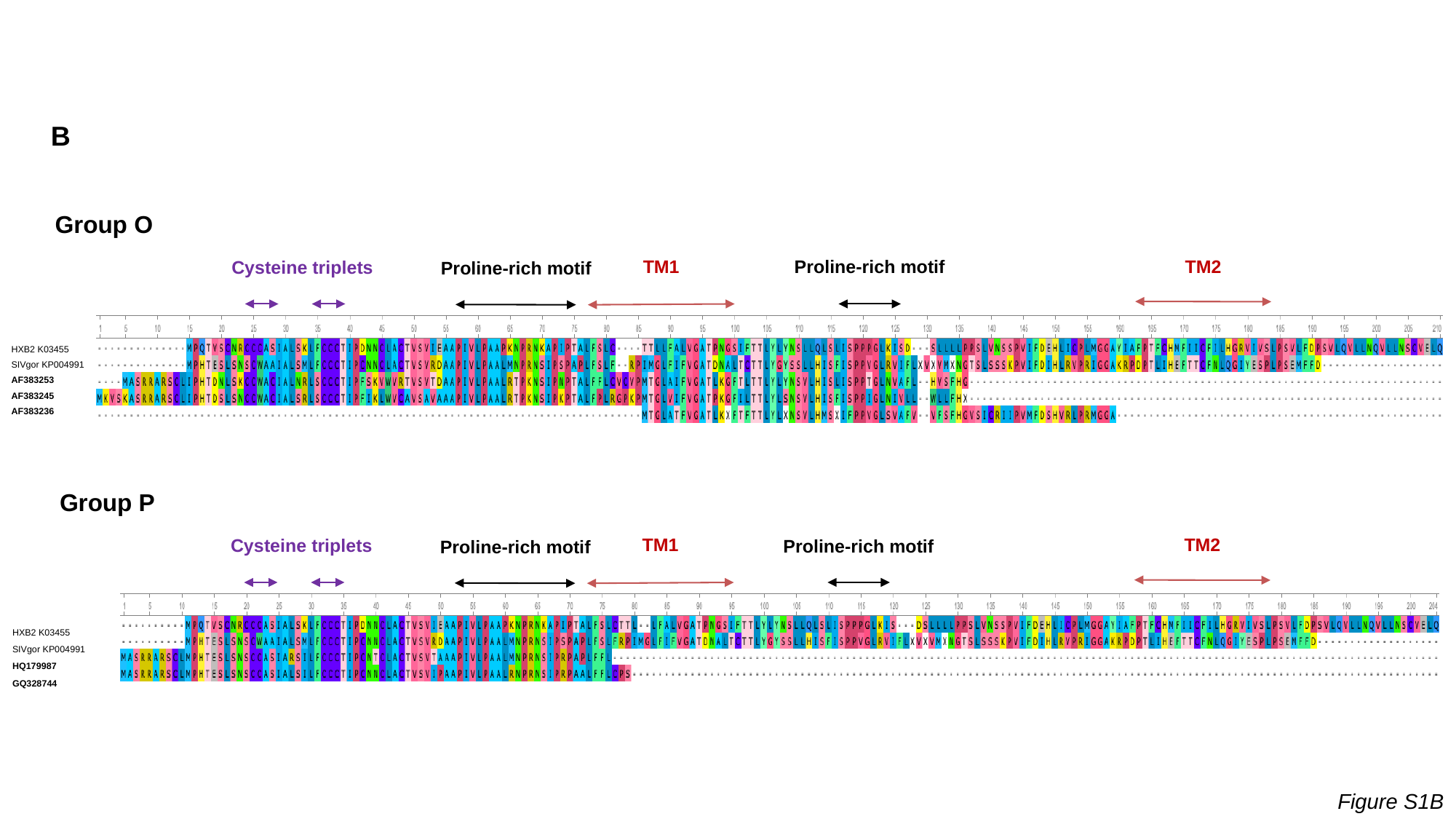

B
Group O
HXB2 K03455
SIVgor KP004991
AF383253
AF383245
AF383236
TM1
Proline-rich motif
TM2
Cysteine triplets
Proline-rich motif
Group P
TM1
TM2
Cysteine triplets
Proline-rich motif
Proline-rich motif
HXB2 K03455
SIVgor KP004991
HQ179987
GQ328744
Figure S1B

## Slide 3
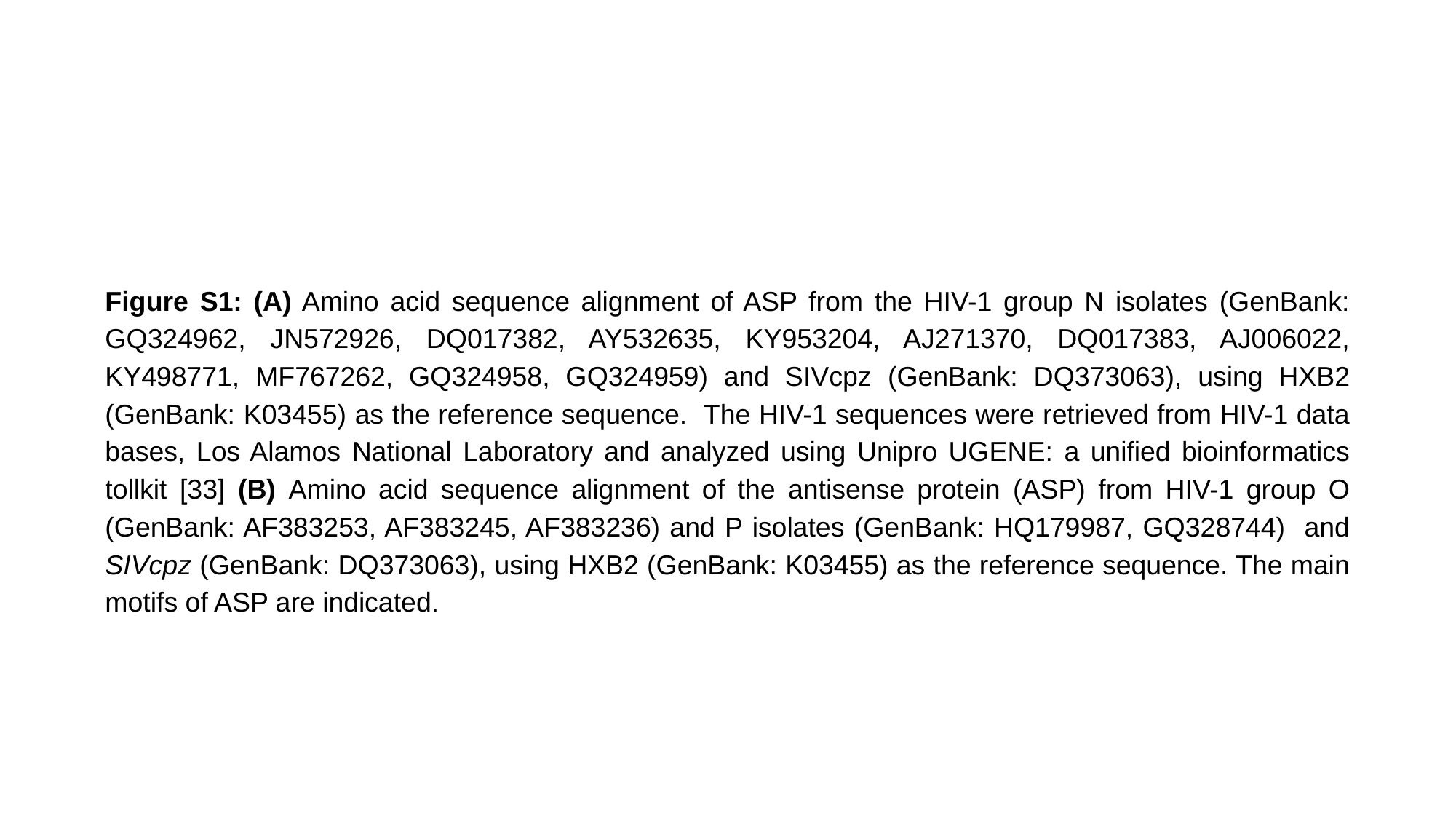

Figure S1: (A) Amino acid sequence alignment of ASP from the HIV-1 group N isolates (GenBank: GQ324962, JN572926, DQ017382, AY532635, KY953204, AJ271370, DQ017383, AJ006022, KY498771, MF767262, GQ324958, GQ324959) and SIVcpz (GenBank: DQ373063), using HXB2 (GenBank: K03455) as the reference sequence. The HIV-1 sequences were retrieved from HIV-1 data bases, Los Alamos National Laboratory and analyzed using Unipro UGENE: a unified bioinformatics tollkit [33] (B) Amino acid sequence alignment of the antisense protein (ASP) from HIV-1 group O (GenBank: AF383253, AF383245, AF383236) and P isolates (GenBank: HQ179987, GQ328744) and SIVcpz (GenBank: DQ373063), using HXB2 (GenBank: K03455) as the reference sequence. The main motifs of ASP are indicated.

## Slide 4
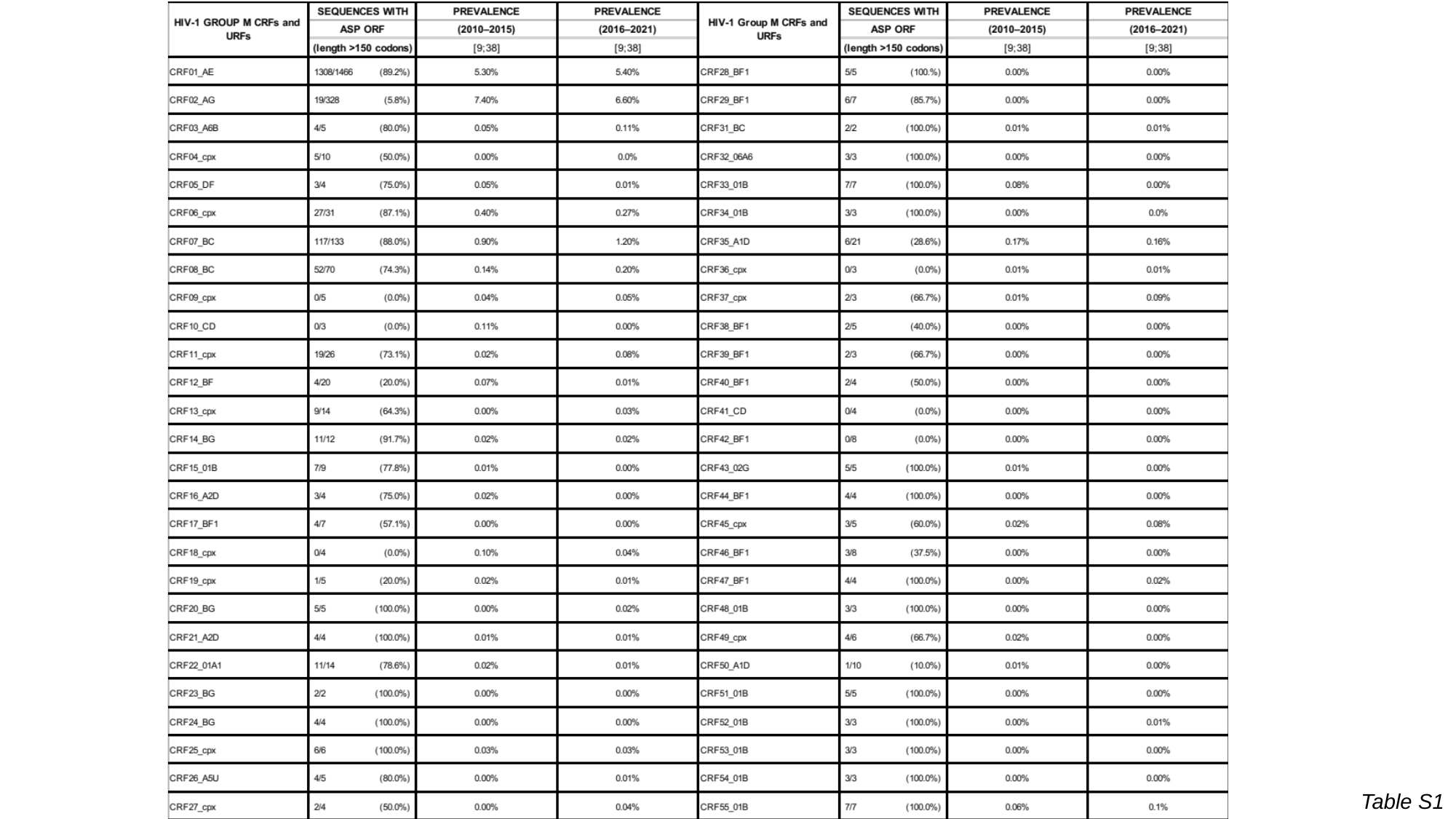

Table S1

## Slide 5
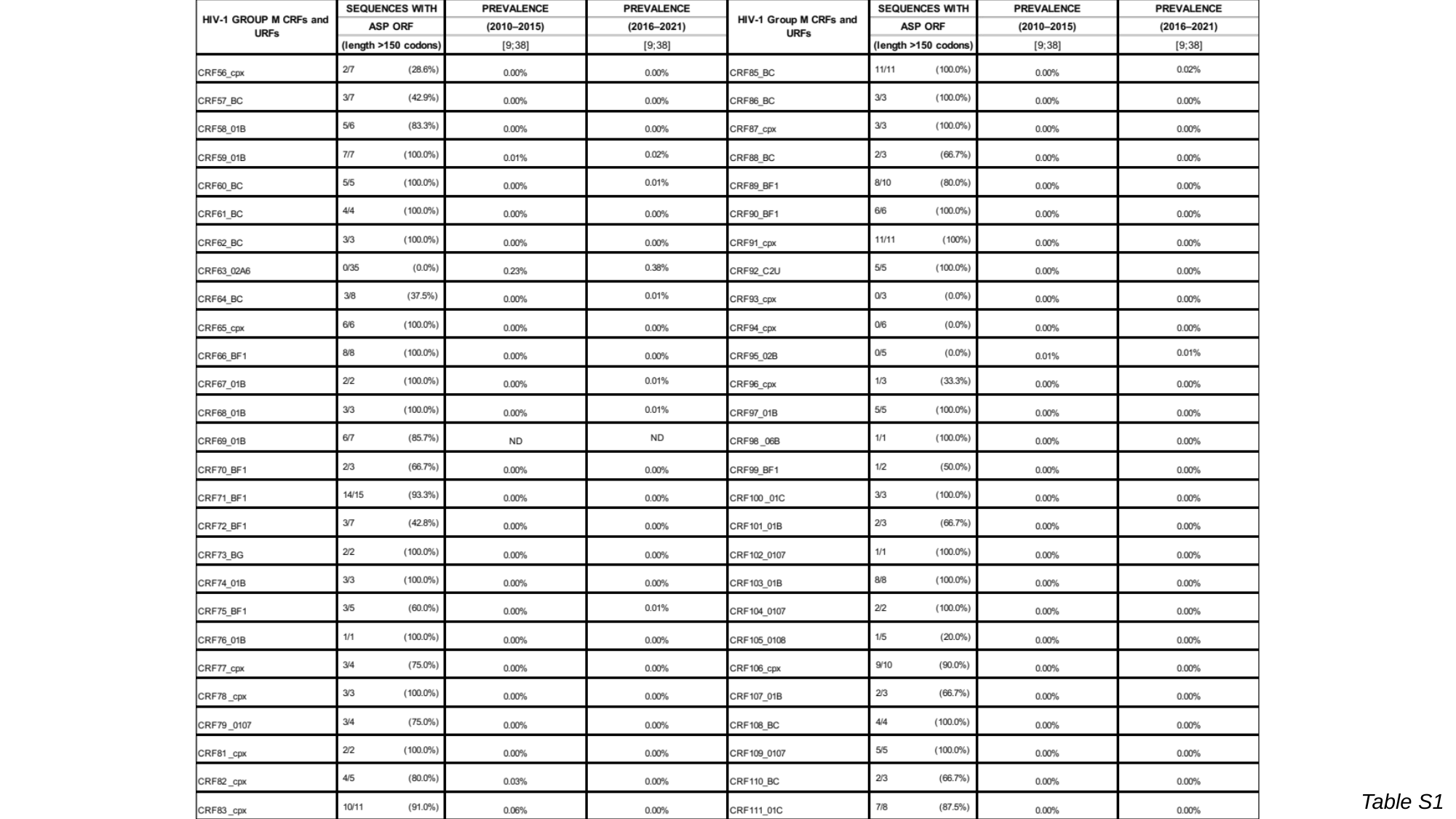

Table S1

## Slide 6
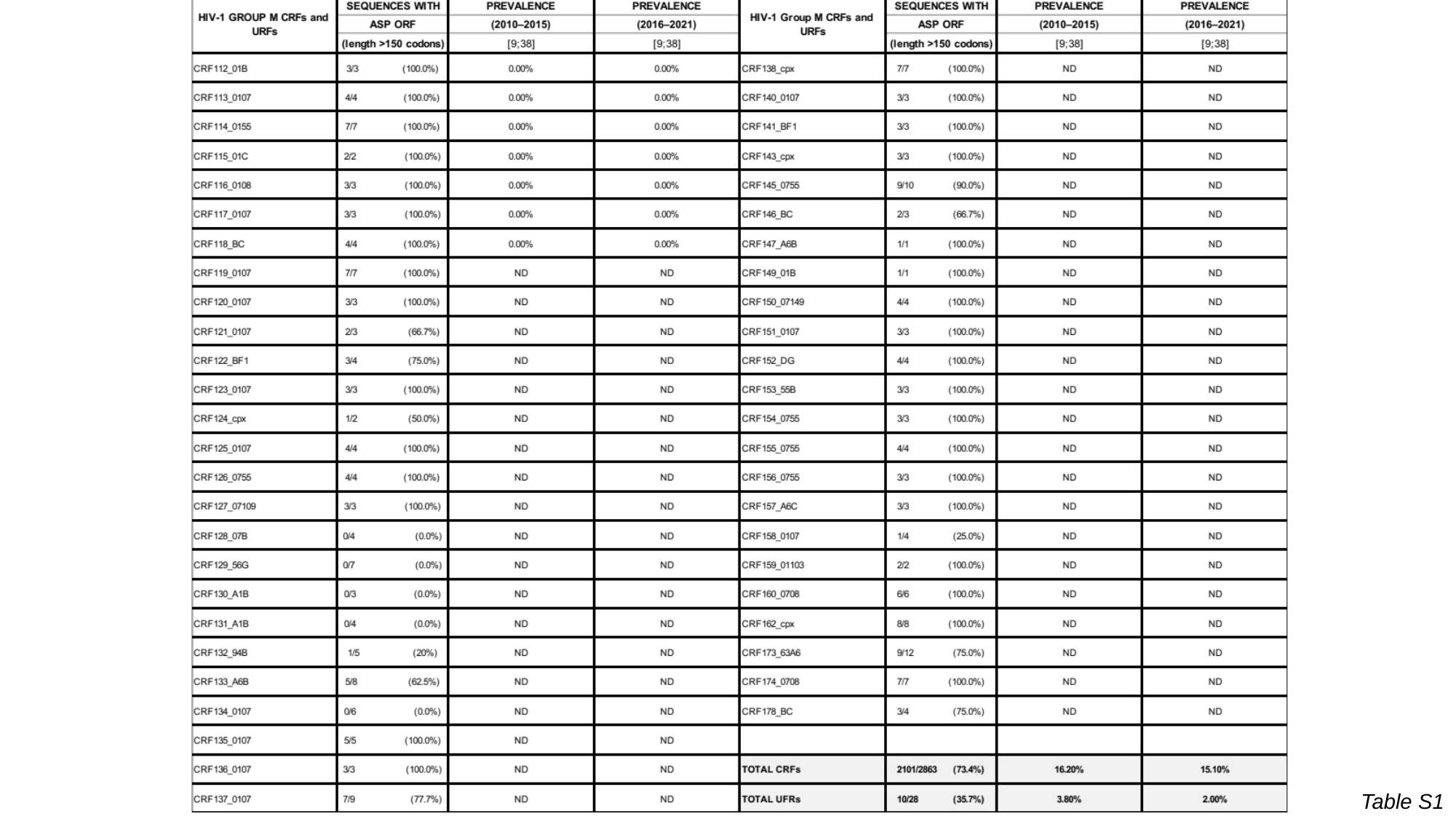

Table S1

## Slide 7
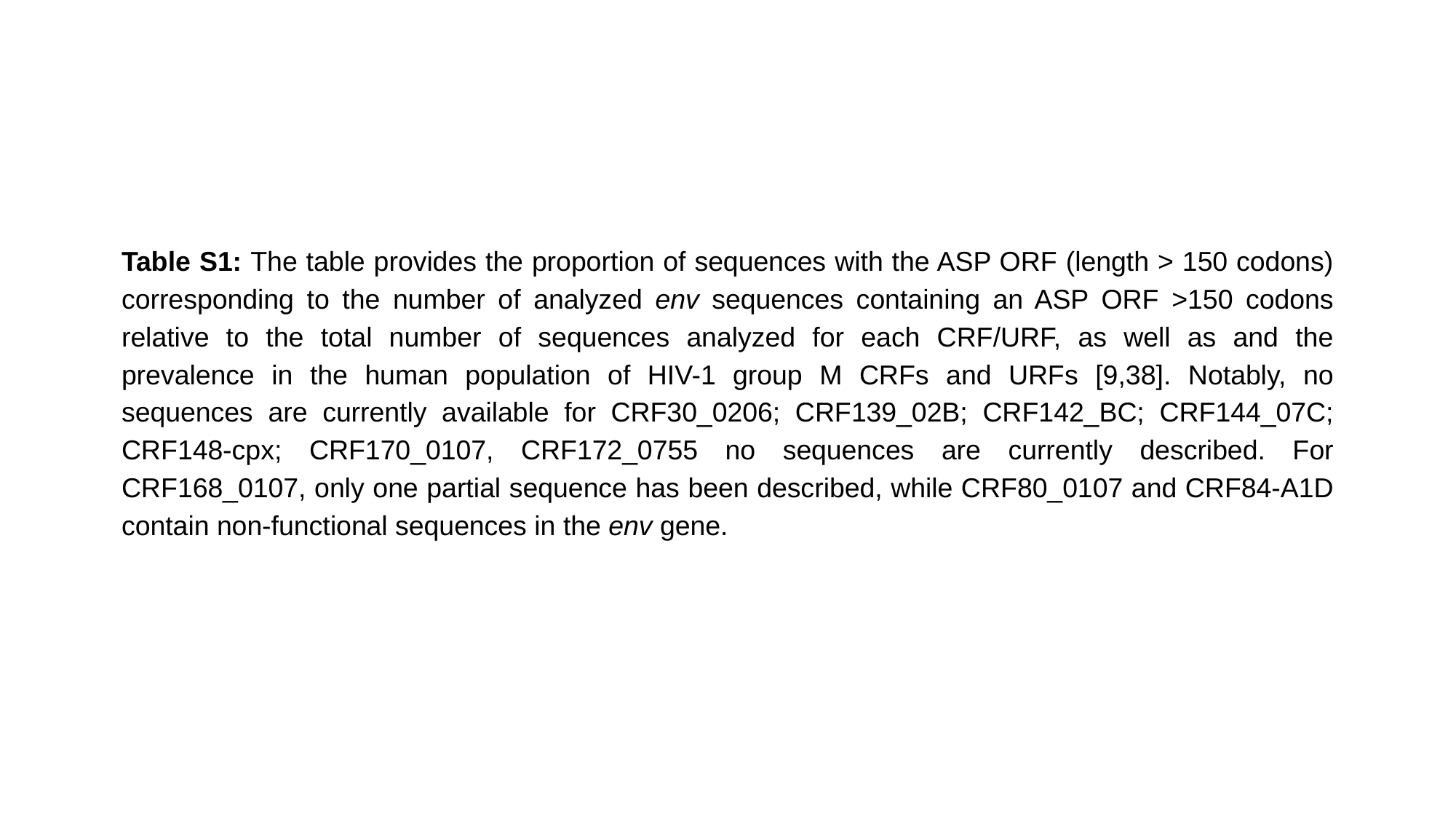

Table S1: The table provides the proportion of sequences with the ASP ORF (length > 150 codons) corresponding to the number of analyzed env sequences containing an ASP ORF >150 codons relative to the total number of sequences analyzed for each CRF/URF, as well as and the prevalence in the human population of HIV-1 group M CRFs and URFs [9,38]. Notably, no sequences are currently available for CRF30_0206; CRF139_02B; CRF142_BC; CRF144_07C; CRF148-cpx; CRF170_0107, CRF172_0755 no sequences are currently described. For CRF168_0107, only one partial sequence has been described, while CRF80_0107 and CRF84-A1D contain non-functional sequences in the env gene.

## Slide 8
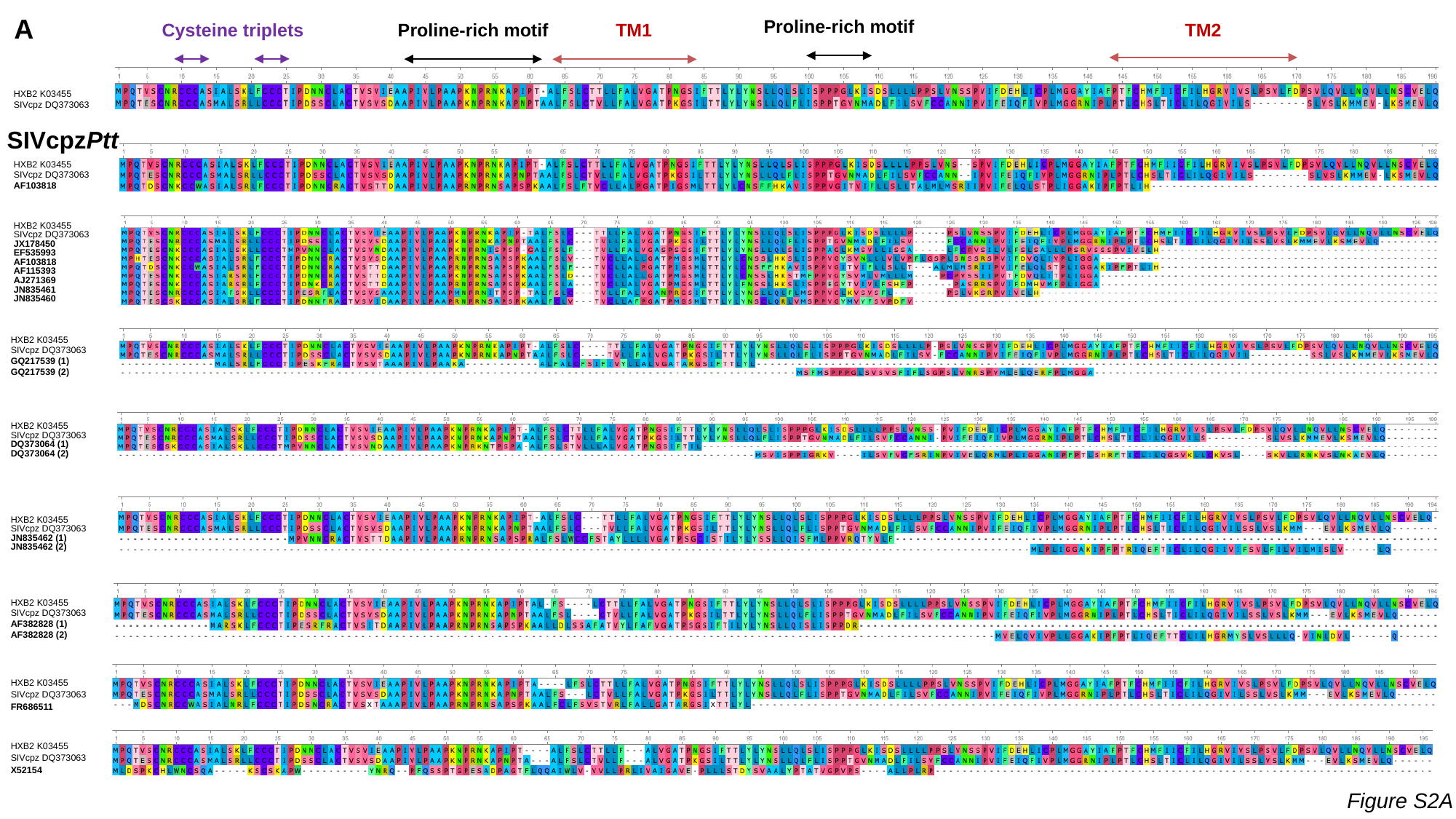

A
Proline-rich motif
Proline-rich motif
TM1
Cysteine triplets
TM2
HXB2 K03455
SIVcpz DQ373063
SIVcpzPtt
HXB2 K03455
SIVcpz DQ373063
AF103818
HXB2 K03455
SIVcpz DQ373063
JX178450
EF535993
AF103818
AF115393
AJ271369
JN835461
JN835460
HXB2 K03455
SIVcpz DQ373063
GQ217539 (1)
GQ217539 (2)
HXB2 K03455
SIVcpz DQ373063
DQ373064 (1)
DQ373064 (2)
HXB2 K03455
SIVcpz DQ373063
JN835462 (1)
JN835462 (2)
HXB2 K03455
SIVcpz DQ373063
AF382828 (1)
AF382828 (2)
HXB2 K03455
SIVcpz DQ373063
FR686511
HXB2 K03455
SIVcpz DQ373063
X52154
Figure S2A

## Slide 9
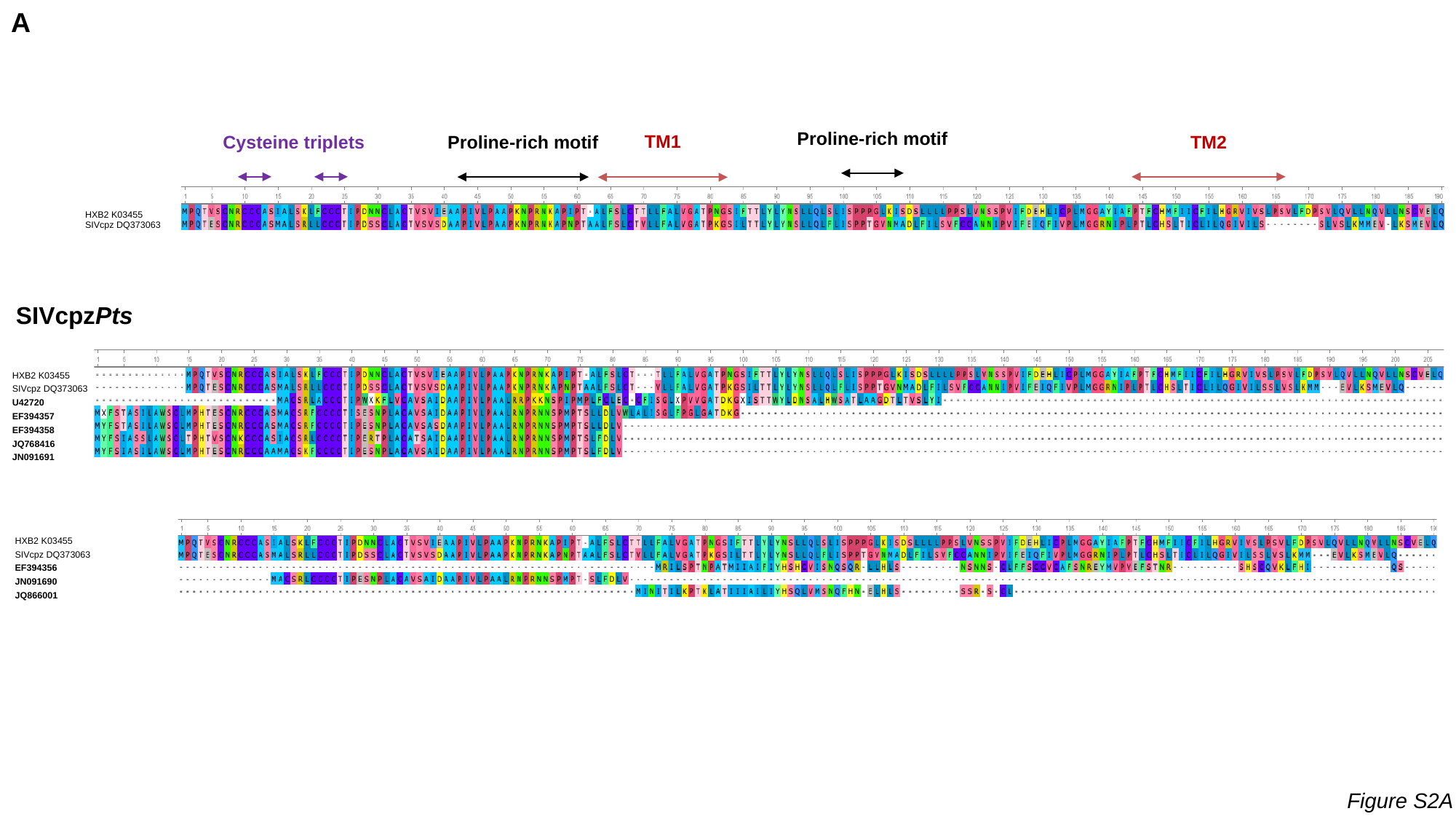

A
Proline-rich motif
TM1
Proline-rich motif
Cysteine triplets
TM2
HXB2 K03455
SIVcpz DQ373063
SIVcpzPts
HXB2 K03455
SIVcpz DQ373063
U42720
EF394357
EF394358
JQ768416
JN091691
HXB2 K03455
SIVcpz DQ373063
EF394356
JN091690
JQ866001
Figure S2A

## Slide 10
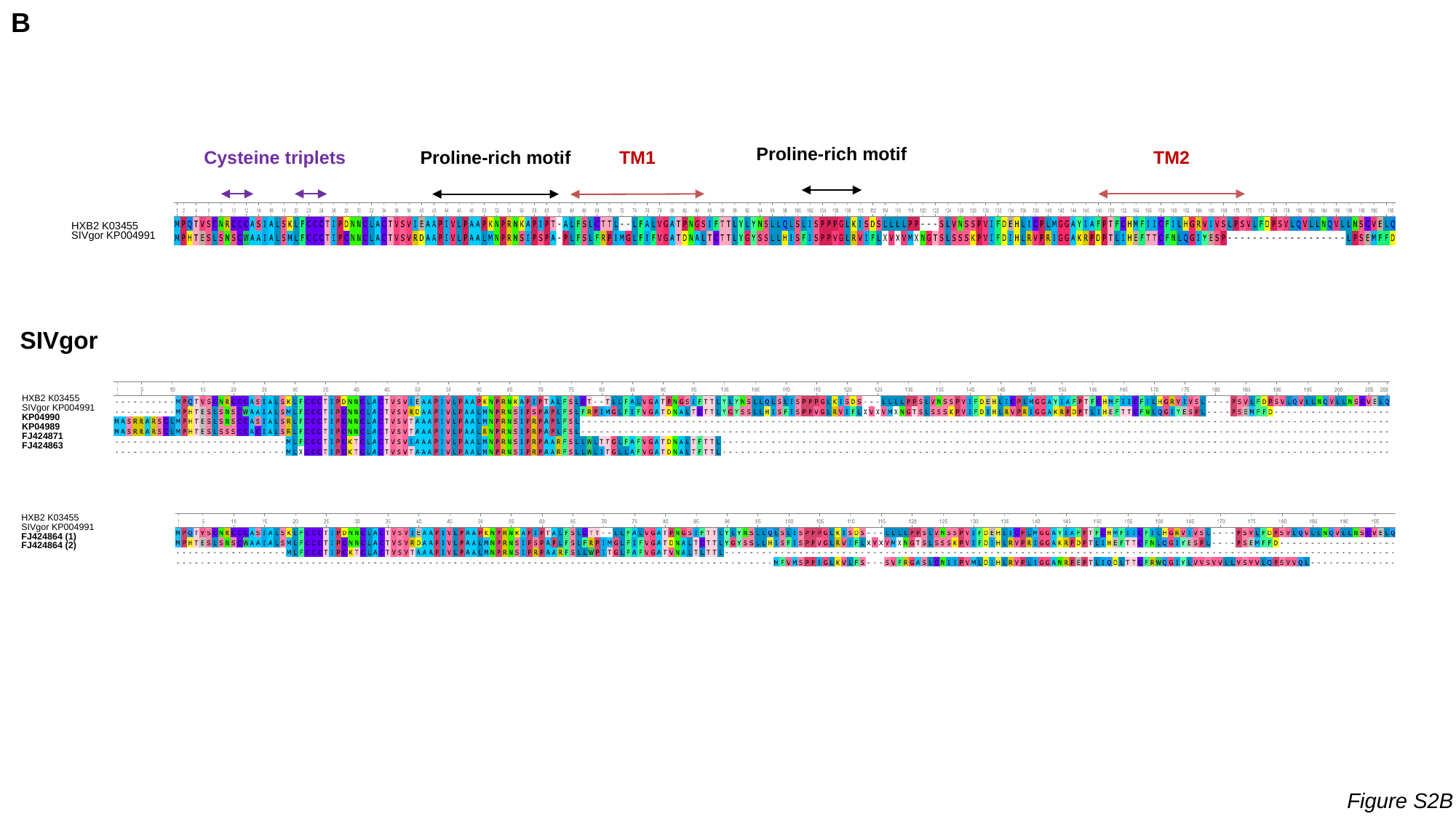

B
Proline-rich motif
TM1
TM2
Proline-rich motif
Cysteine triplets
HXB2 K03455
SIVgor KP004991
SIVgor
HXB2 K03455
SIVgor KP004991
KP04990
KP04989
FJ424871
FJ424863
HXB2 K03455
SIVgor KP004991
FJ424864 (1)
FJ424864 (2)
Figure S2B

## Slide 11
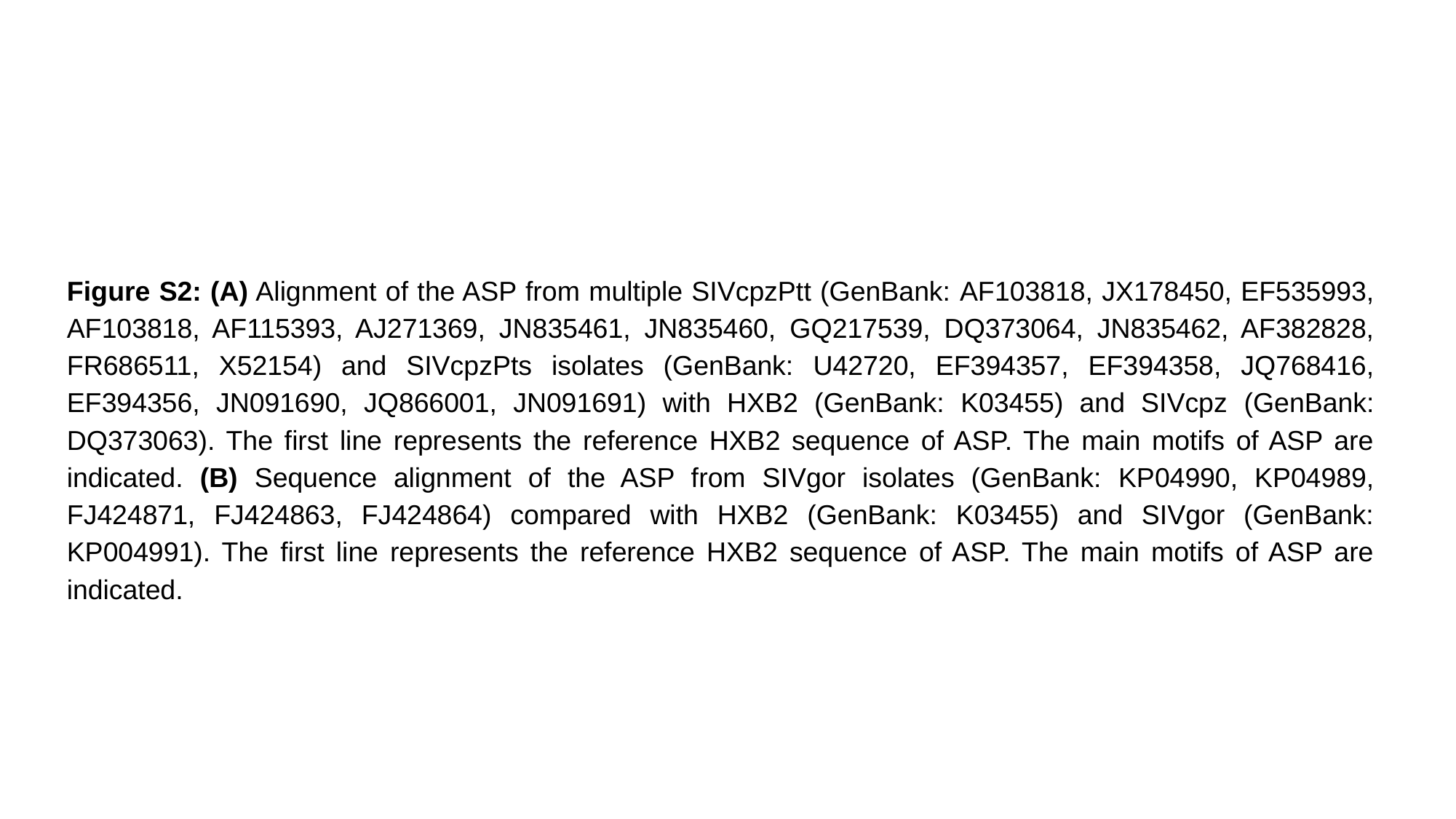

Figure S2: (A) Alignment of the ASP from multiple SIVcpzPtt (GenBank: AF103818, JX178450, EF535993, AF103818, AF115393, AJ271369, JN835461, JN835460, GQ217539, DQ373064, JN835462, AF382828, FR686511, X52154) and SIVcpzPts isolates (GenBank: U42720, EF394357, EF394358, JQ768416, EF394356, JN091690, JQ866001, JN091691) with HXB2 (GenBank: K03455) and SIVcpz (GenBank: DQ373063). The first line represents the reference HXB2 sequence of ASP. The main motifs of ASP are indicated. (B) Sequence alignment of the ASP from SIVgor isolates (GenBank: KP04990, KP04989, FJ424871, FJ424863, FJ424864) compared with HXB2 (GenBank: K03455) and SIVgor (GenBank: KP004991). The first line represents the reference HXB2 sequence of ASP. The main motifs of ASP are indicated.

## Slide 12
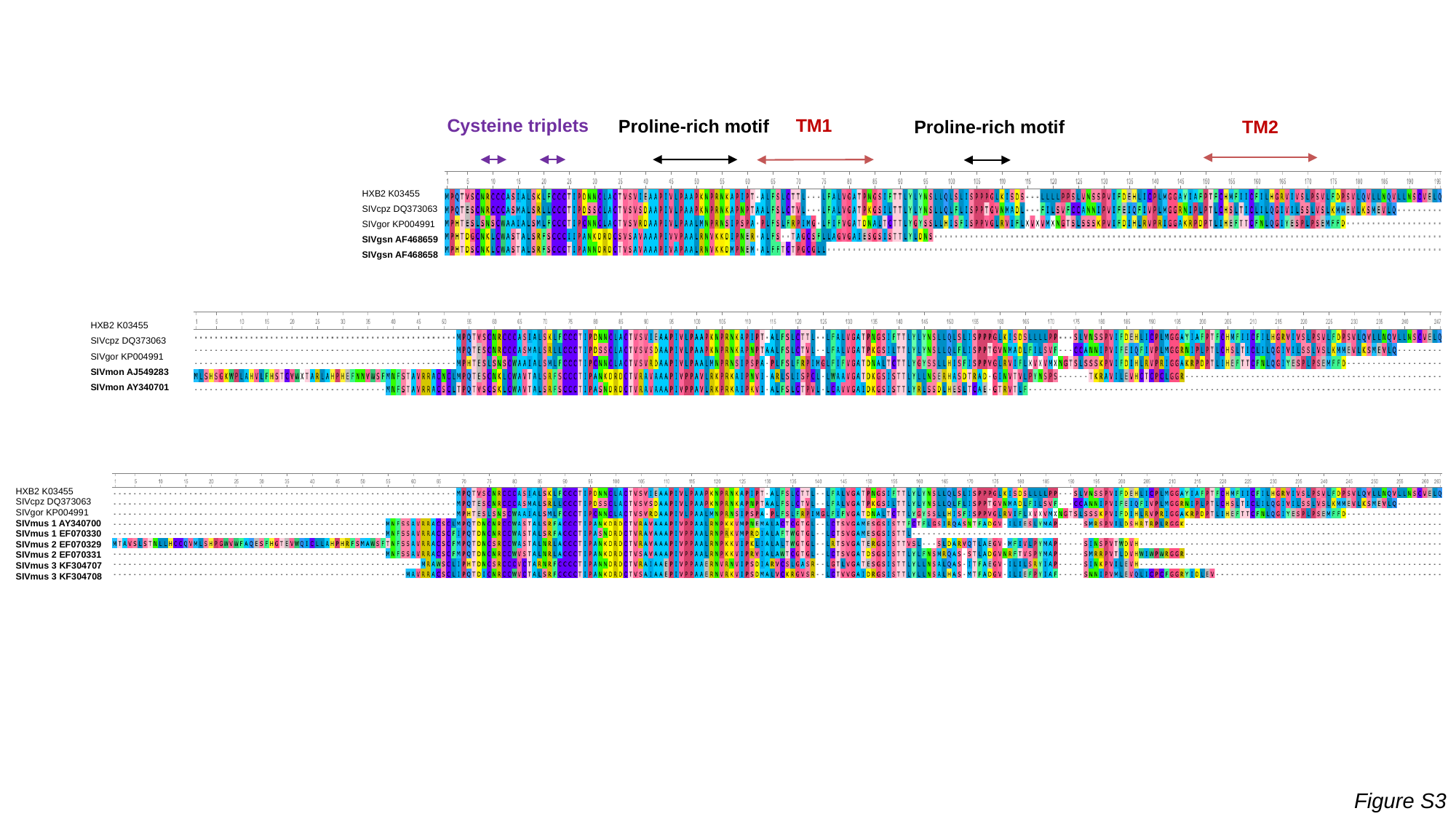

TM1
Cysteine triplets
Proline-rich motif
TM2
Proline-rich motif
HXB2 K03455
SIVcpz DQ373063
SIVgor KP004991
SIVgsn AF468659
SIVgsn AF468658
HXB2 K03455
SIVcpz DQ373063
SIVgor KP004991
SIVmon AJ549283
SIVmon AY340701
HXB2 K03455
SIVcpz DQ373063
SIVgor KP004991
SIVmus 1 AY340700
SIVmus 1 EF070330
SIVmus 2 EF070329
SIVmus 2 EF070331
SIVmus 3 KF304707
SIVmus 3 KF304708
Figure S3

## Slide 13
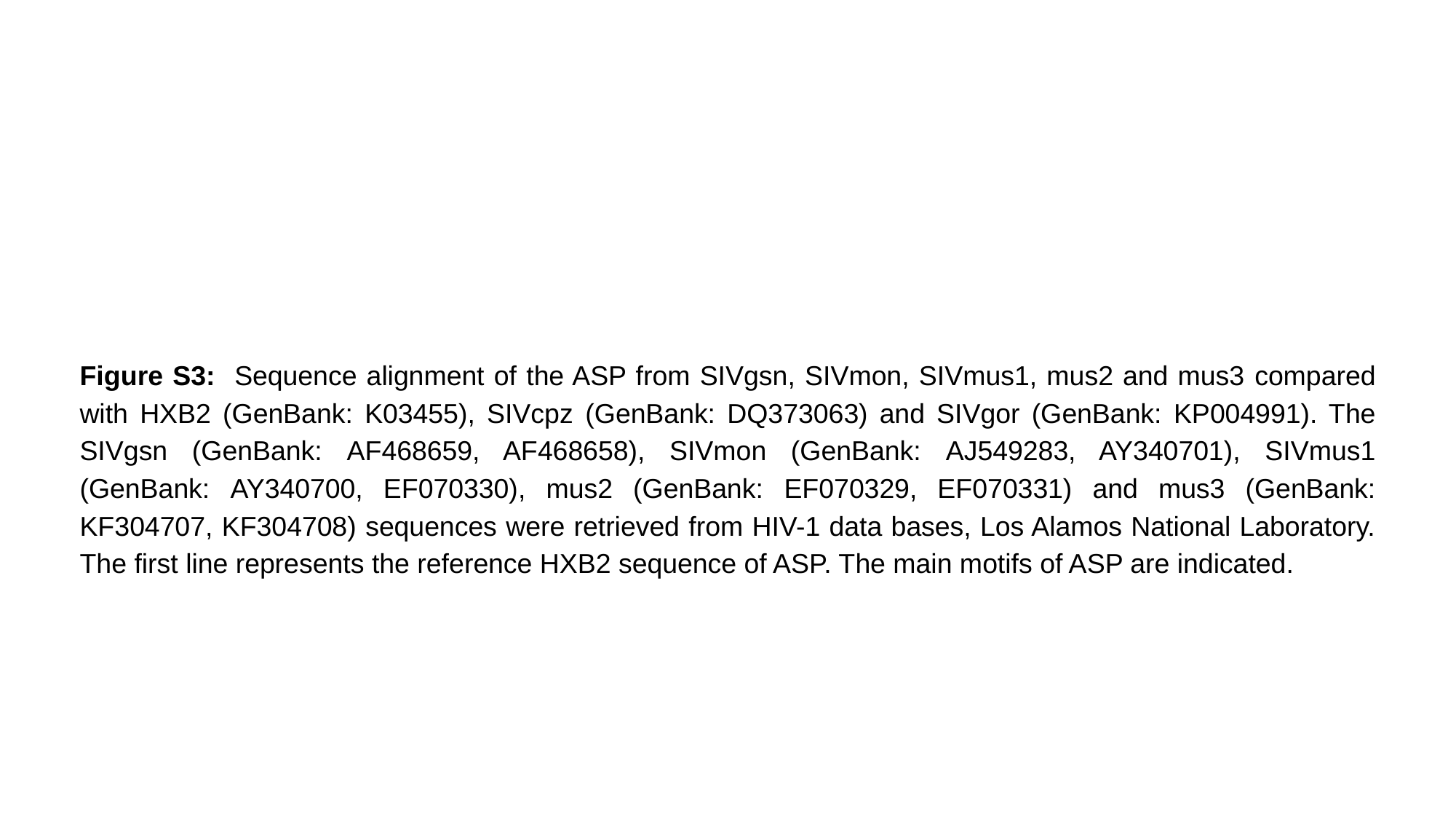

Figure S3: Sequence alignment of the ASP from SIVgsn, SIVmon, SIVmus1, mus2 and mus3 compared with HXB2 (GenBank: K03455), SIVcpz (GenBank: DQ373063) and SIVgor (GenBank: KP004991). The SIVgsn (GenBank: AF468659, AF468658), SIVmon (GenBank: AJ549283, AY340701), SIVmus1 (GenBank: AY340700, EF070330), mus2 (GenBank: EF070329, EF070331) and mus3 (GenBank: KF304707, KF304708) sequences were retrieved from HIV-1 data bases, Los Alamos National Laboratory. The first line represents the reference HXB2 sequence of ASP. The main motifs of ASP are indicated.
